# Nanoparticle-Induced Interfacial Director Straining Enables Ultrasensitive Molecular Detection at Microfluidic Liquid Crystal Interfaces

**DOI:** 10.1021/acsami.6c02354

**Published:** 2026-07-16

**Authors:** Pouriya Esmaeilzadeh, Emre Bukusoglu

**Affiliations:** † Department of Chemical Engineering, Middle East Technical University, Dumlupınar Bulvarı No.1, Çankaya, Ankara 06800, Türkiye; ‡ Department of Micro and Nanotechnology, Middle East Technical University, Dumlupınar Bulvarı No.1, Çankaya, Ankara 06800, Türkiye

**Keywords:** liquid crystals, nanoparticles, microfluidics, ultrasensitive sensors, aqueous pollutants

## Abstract

Detecting aqueous trace pollutants is a challenge in tracking environmental and public health. We report nanoparticle-decorated liquid crystal (LC) soft interfaced microfluidic platforms for detecting trace-level aqueous analytes. Silica nanoparticles functionalized with alkyl-terminated silanes were used to decorate the LC–aqueous interfaces and induce LC strain. The response of the LC flow sensor to various analytes, including industrial dyes, pharmaceuticals, and persistent chemicals, was investigated through the optically observable LC ordering transitions. The microfluidic results, supported by nanoparticle-integrated LC droplet-based sensors, showed a limit of detection (LOD) of 0.1 ppb with LC interfaces decorated with concentrated nanoparticles. Lowering the interfacial nanoparticle loading resulted in sensors achieving an impressive 4 orders of magnitude reduction in the LOD. While LC droplet-based sensors were unable to reach this sensitivity limit, such ultralow responses were strikingly observed in microfluidic sensor platforms by leveraging the interface geometry and localized straining at LC interfaces induced by nanoparticle positioning. We show that the sensors demonstrate selectivity for analytes characterized by aromatic structures. These features hold potential for various applications, including continuous tracking of micropollutants and medical diagnostics.

## Graphical abstract

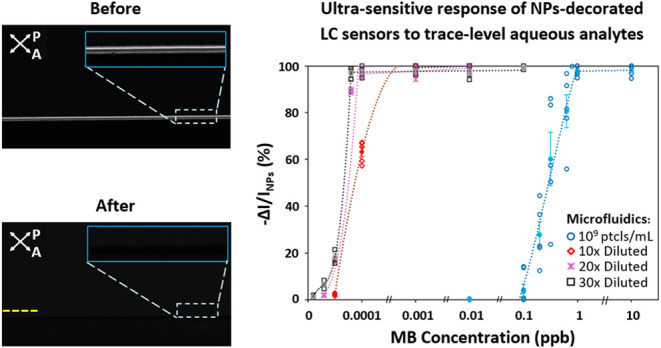

## 1 Introduction

The detection of trace-level concentrations of species in an aqueous medium (ng/L to μg/L range) is of paramount importance due to global concerns regarding the unfavorable impacts of micropollutants on health and the ecosystem.^1–3^ This raises an apparent demand for sensor platforms that can facilitate straightforward and continuous measurements while being tailorable and adaptable for a diverse range of chemical species. The comprehensive investigations into liquid crystals (LCs), specifically the nematic LC phase, have revealed their considerable potential for label-free sensing applications.^4–6^ The sensing mechanism in chemical- and biological sensors utilizing LCs as signal reporters relies on the existence of an interfacial stimuli, including electrostatic interaction, physical adsorption, or chemical binding, to perturb the interfacial orientational ordering of the LCs.^7,8^ Such interfacial ordering transition of LCs transforms into optical signals owing to the long-range orientational ordering and inherent optical anisotropy of the LCs, allowing for easy visual rendering under a polarized optical microscope.^9^ Thus, they function as an “optical amplifier” of the interfacial interactions, responding to external stimuli through ordering transitions over a short time period.^10–12^ Recent studies regarding LCs in responsive systems have primarily concentrated on their interfacial phenomena in aqueous media, namely their interactions with analytes in aqueous phase for sensing objectives, the synthesis of nanostructured materials, and their responses to chemical or mechanical stresses at the LCs interfaces.^13–18^ Despite promising reported results that could be leveraged for critical applications, the findings have predominantly been constrained to stagnant LC systems, which significantly limit their use in continuous operation contexts. However, the advancement of the encouraging achievements in stagnant LC systems can be realized through the integration of LCs into automated flow systems for pivotal applications, including point-of-care and marker-based diagnostics, early warning/prognosis evaluation of disease, environmental/public health monitoring, and real-time industrial control, among others, which demand high-throughput, continuous, or periodic analysis and rapid action. These features can be unlocked when LC-based sensors meet microfluidics.

Microfluidics enable the accurate control and manipulation of fluids within microscale channels, facilitating the miniaturization of intricate laboratory processes such as detection, separation, and sample preparation into monolithic, portable systems.^19,20^ Lab-on-a-chip systems constructed in microfluidics have emerged as an effective tool in the development of sensing platforms, offering transformative advantages like simple integration, drastically reduced detection time, ultralow sample consumption, efficient mass transfer, and field-deployable portability.^21^ However, limitations in sensitivity and selectivity, such as low detectable signals and poor signal-to-noise ratios, are ongoing challenges in microfluidic systems.^22–25^ The integration of microfluidics with advanced/soft functional materials, including nanomaterials^24,26–29^ and LCs, holds promise to address these restrictions and promote the performance of microfluidic sensors.

Nanomaterials demonstrate distinctive properties, including tunable interfacial chemistry, high surface-to-volume ratio, and enhanced signal amplification, positioning them as attractive candidates for improving sensor performance, including sensitivity and selectivity to detect trace-level concentrations.^23,30^ Recently, we have demonstrated that nanoparticle-assisted LC droplet-based sensors enable the sensitive detection of species that do not directly induce an ordering transition at the LC–water interfaces.^31^ This capability, which was achieved by employing silica nanoparticles functionalized with mixed monolayers of carboxyl- and alkyl-terminated silane ligands, expands the range of analytical targets that can be effectively analyzed. Other recent studies also showed that ligand chemistry on nanoparticles plays a crucial role in interfacial interactions, orientational ordering of LC at interfaces, and sensor functionality. As a case in point, it was shown that amphiphilic gold nanoparticles functionalized with PEG-thiol and hexadecanethiol stabilized LC-in-water emulsions with enhanced colloidal stability and tunable sensitivity to surfactants, improving sensor longevity and selectivity.^32^ Oñate-Socarras et al. developed a biosensing platform using SiO_2_/C*_n_* TAB complex to stabilize thermotropic LC droplets, demonstrating that the adsorption of phospholipid vesicles induced a sensitive optical transition influenced by the ligand’s tail length and lipid structure.^33^ Ning et al. also indicated that the biphenylalkyl ligand shells on nanoparticles can be induced to adopt anisotropic conformations by the LC environment, which aligns the nanoparticles with the LC director and allows external fields to manipulate their orientation, thereby enhancing tunability in LC-based systems.^34^ Hence, the synergy between ligand chemistry and LC ordering advances the design of responsive nanomaterials for sensing and photonic applications.

The formation of stable “soft” LC–aqueous interfaces enables the monitoring of spatiotemporal changes in the ordering of LC phases owing to the subtle response of the LCs to the chemical and mechanical stimuli at their aqueous interfaces. Such a platform not only facilitates the integration of LCs into continuous systems but also paves the way for enhancing the capabilities of the LC-based responsive systems, including automated flow devices for advanced analytical techniques. Recently, we succeeded in stabilizing the aqueous interfaces and quantifying the structural transitions in flowing nematic LCs confined in microfluidic channels with accessible LC–aqueous soft interfaces.^35,36^ However, developing such a platform to attain reliable high-throughput responsive systems that can be integrated with advanced materials for cutting-edge sensing purposes, including the detection of solutes in aqueous phases that do not readily cause a direct ordering transition at the LC–aqueous interface, has yet to be achieved.

In this study, we investigate the fabrication of nanoparticle-integrated LC-based microfluidic sensors within microchannels featuring stable soft interfaces between thermotropic nematic 4-cyano-4′-pentylbiphenyl (5CB, an anisotropic oil) and aqueous phases, to detect low-concentration analyte molecules from the aqueous phase. To this end, we decorated the LC–aqueous interface with concentrated, functional fluorescent silica nanoparticles and investigated the response characteristics of the nanoparticle-decorated LC flow sensors against a range of industrially or environmentally relevant chemical species. We revealed how the structuring of the adsorbed nanoparticles at the LC–aqueous interface can dramatically affect the sensitivity, such that the LOD of the nanoparticle-concentrated interface was measured as low as 0.1 ppb, while diluted nanoparticle-interfaced counterparts exhibited a substantial decrease in the LOD by 4 orders of magnitude (ppt-level). We found that the significant enhancement in LOD was directly related to the positioning of the nanoparticles at the interfaces, forming local anchoring heterogeneities (thus straining) that cause a favorable anchoring transition at ppt concentration levels. We demonstrate that the functionalized nanoparticles exhibit favorable selectivity performance in the detection of various analytes, particularly those with aromatic structures. The developed sensor is expected to achieve widespread application in automated flow systems, including the tracking of trace pollutants in aqueous environments, while offering a path for future advancements in the field.

## 2 Results and Discussion

Within a microfluidic channel, a stable and well-defined soft “virtual wall” interface between 5CB (Figure 1a) and an aqueous phase was maintained, spanning 1.5 cm in length and 14 μm in depth. This was achieved by employing a selective surface functionalization strategy that leverages the wetting properties of the immiscible thermotropic room-temperature nematic LC (5CB) and aqueous phases. Specifically, one longitudinal side of the microchannel was rendered hydrophobic through a flow-focusing of an aqueous solution of DMOAP, while the remaining side was left uncoated to retain its hydrophilic nature.^35,36^ This spatially patterned wettability enabled the preferential filling of the DMOAP-coated side of the channel with 5CB upon cointroduction with water at controlled inlet pressures, thereby generating a microfluidic system featuring a robust 5CB–water interface. The stability of the “virtual wall” separating the coflowing 5CB and water was confirmed through brightfield and polarized light microscopy, as shown in Figure 1d. The flow of 5CB and aqueous phases during the experiments was all horizontal from the top view (*x*–*y* plane). The dark optical texture observed under polarized light indicated a uniform perpendicular alignment of the 5CB director field relative to the imaging plane, driven by the homeotropic anchoring at the DMOAP-functionalized interfaces and minimal flow-induced distortion of the LC director field. Additionally, the 5CB–water interface exhibited planar anchoring, consistent with the dark appearance observed in the polarized micrographs. The fluorescence micrographs appeared dark, as pure water was used in the aqueous phase. The LC director profile across the channel is depicted in the cross-sectional schematic in Figure 1f (top).

**Figure 1:**
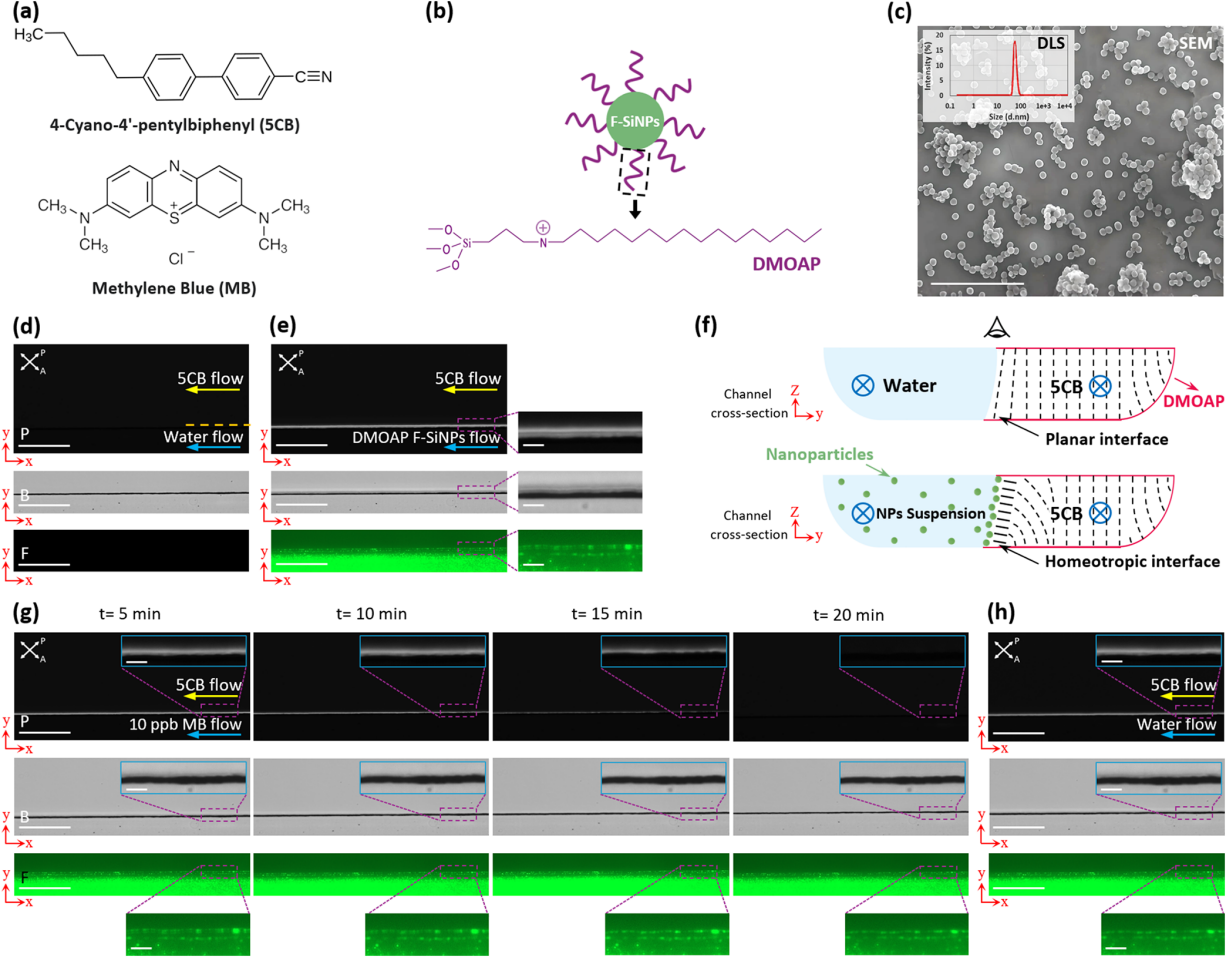
Chemical structure of (a) 5CB, MB, and (b) DMOAP employed for functionalizing the F-SiNPs. (c) SEM micrograph of the F-SiNPs. The inset shows the corresponding hydrodynamic particle size measured by DLS. The polarized (P), brightfield (B), and fluorescence (F) microscopy images of (d) 5CB–water and (e) 5CB–aqueous DMOAP F-SiNPs (pH = 2) systems in a channel during weak flow, representing the planar and homeotropic anchoring of 5CB–aqueous interface, respectively. (f) Schematic illustration of the cross-sectional nematic director configuration maintained in microfluidic channels featuring planar or homeotropic aqueous interfaces. (g) Response of the nanoparticle-decorated LC flow sensor (∼10^9^ particles/mL of DMOAP F-SiNPs) to 10 ppb MB concentration over time, showing the orientational ordering transition of the interface from homeotropic to planar anchoring upon adsorption of MB molecules to the interface in 20 min. (h) Rinsing the channel with water at the end of the sensing process reveals that the system is reversible, allowing the interface to change to the homeotropic state as the MB molecules are removed. Scale bars: 1 μm for the SEM micrograph; 100 μm for the P, B, and F micrographs and 10 μm for their zoomed parts.

Figure 1b schematically illustrates the structure of fluorescent silica nanoparticles functionalized with DMOAP (henceforth referred to as DMOAP F-SiNPs). The scanning electron microscope (SEM) micrograph of DMOAP F-SiNPs presented in Figure 1c revealed that the synthesized nanoparticles exhibit spherical morphology, with an average particle size of around 70 ± 6 nm. The measured size was in significant agreement with the average hydrodynamic diameter of particles determined to be 72 ± 3 nm through dynamic light scattering (DLS) analysis (Figure 1c, inset). By introducing the aqueous DMOAP F-SiNPs suspension with pH = 2 to the system, the 5CB–aqueous phase interface was shifted to homeotropic anchoring upon the adsorption of nanoparticles to the interface. Previously, we showed that DMOAP F-SiNPs exhibit suitable stability across a range of acidic pH levels from 2 to 6. This stability was evidenced by ζ-potential values of 50–60 mV within this pH range.^37^ Additionally, to ensure optimal dispersion of the nanoparticle suspension, adequate tip sonication was performed immediately before use. Polarized optical microscopy confirmed the anchoring transition, showing a bright appearance at the 5CB–aqueous interface after the microchannel treatment with the nanoparticle suspension (Figure 1e), as illustrated in the schematic configuration in Figure 1f (bottom). Such homeotropic anchoring of 5CB was also reflected as a dark halo above the interface in the brightfield image. The presence of DMOAP F-SiNPs and their decoration at the interface of 5CB and the aqueous phase was validated using fluorescence microscopy (Figure 1e).

Recently, it was demonstrated that nanoparticle-assisted LC droplet sensors are promising in the determination of the aqueous soluble analytes that do not cause a direct ordering transition at the LC–water interfaces.^31^ While we were inspired by such work, our approach was distinct and more pragmatic. We first developed the nanoparticle-decorated LC-aqueous interface within the microfluidic platform, and subsequently introduced the analytical species to ascertain their concentrations in the aqueous medium. This differed from the typical method in the literature, which equilibrates nanoparticles and analytes before exposing them to the LC droplets interface. The 5CB–water interface was decorated with ∼10^9^ particles/mL of DMOAP F-SiNPs suspension, followed by the injection of a 10 ppb aqueous solution (as a demonstration) of Methylene Blue (MB) (Figure 1a) into the channel. The experiment was conducted at low bulk and interfacial shear stress (0.1 Pa) with equal 5CB and aqueous phase inlet pressures P_5CB_ = P_Aq._ = 5 mbar,^36^ to avoid the free displacement of nanoparticles owing to the high shear stress and enable effective adsorption of dye molecules onto the nanoparticles. Under these conditions, we measured the interfacial velocity to be <1 μm/s, confirming their insignificant displacement. As is evident from the polarized light micrographs in Figure 1g, the bright appearance of the nanoparticle-decorated 5CB–aqueous interface assumed a darker appearance over time, indicating the transition of 5CB from a homeotropic to planar interfacial anchoring. The analyte sensing process was accomplished in 20 min for a 10 ppb concentration of MB. The brightfield micrographs also indicated that the dark halo at the interface faded over time due to this transition. The fluorescence micrographs revealed that the adsorbed nanoparticles at the interface did not show a significant displacement during the sensing process (bottom row of Figure 1g). Hence, the interfacial ordering transition of 5CB from homeotropic to planar anchoring was attributed to the adsorption of MB molecules to the nanoparticle-decorated 5CB–aqueous interface. Rinsing the channel with pure water at the end of the “sensing process” demonstrated that the system was reversible (Figure 1h). This means that while the nanoparticles maintained their position at the interface, the adsorbed MB molecules can be desorbed from the nanoparticle-decorated 5CB interface and washed away with the water flow. Consequently, 5CB transitioned from a planar to a homeotropic interfacial orientation, resulting in the 5CB–water interface appearing bright under the polarized optical microscope, as illustrated in Figure 1h.

A series of experiments were conducted to demonstrate that the homeotropic 5CB–aqueous interface state achieved in Figure 1e was specifically assigned to the effective appearance of the nanoparticles at the interface. As stated earlier, an aqueous DMOAP F-SiNPs suspension with pH = 2 was employed to decorate the 5CB–water interface with the nanoparticles. The application of such an acidic pH was necessitated by the observation that using the nanoparticle suspension at its initial pH of 7 resulted in the rapid occlusion of the channel inlet on the aqueous side, which consequently led to the swift termination of the experimental procedures (Figure 2a). This blockage occurred owing to the strong affinity of the positively charged DMOAP F-SiNPs to adsorb onto either the inherently negatively charged glass or the PDMS that was bonded to it through the oxygen plasma treatment.^38,39^ At pH of 2, although the interfacial charge of the DMOAP F-SiNPs remained positive, the silanol groups on the glass and PDMS surfaces became protonated, reducing the attraction of nanoparticles.^38,40^ To investigate the effect of pH on the 5CB–aqueous interface, we introduced water with pH = 2 to a microchannel. As shown in Figure 2b, such acidic water could induce a slight shift in the 5CB interfacial anchoring toward homeotropic, determined by a narrow, faint bright line at the 5CB–water interface. However, this bright appearance was diminished following the injection of water with neutral pH, restoring the interface to a planar state. As another control experiment, a 10 ppb MB flow was injected into a channel (Figure 2c). As anticipated, the planar anchoring of 5CB at the interface remained consistent because the water-soluble MB does not induce an observable ordering transition at the LC-water interface. Finally, to indicate that the nanoparticles predominantly facilitate the detection of MB molecules at the 5CB–water interface (as considered in Figure 1g), the interface was decorated with DMOAP F-SiNPs in an aqueous suspension with pH = 2. Subsequently, neutral pH water flow was introduced into the channel. In contrast to the experiment done with water at pH = 2 (see Figure 2b), the homeotropic state of the interface and its bright appearance remained unchanged following the injection of water with a neutral pH. Therefore, it can be inferred that the adsorbed nanoparticles at the 5CB–water interface serve as a crucial mediator for detecting species in the aqueous medium within microfluidic sensors through facilitating LC anchoring transitions.

**Figure 2:**
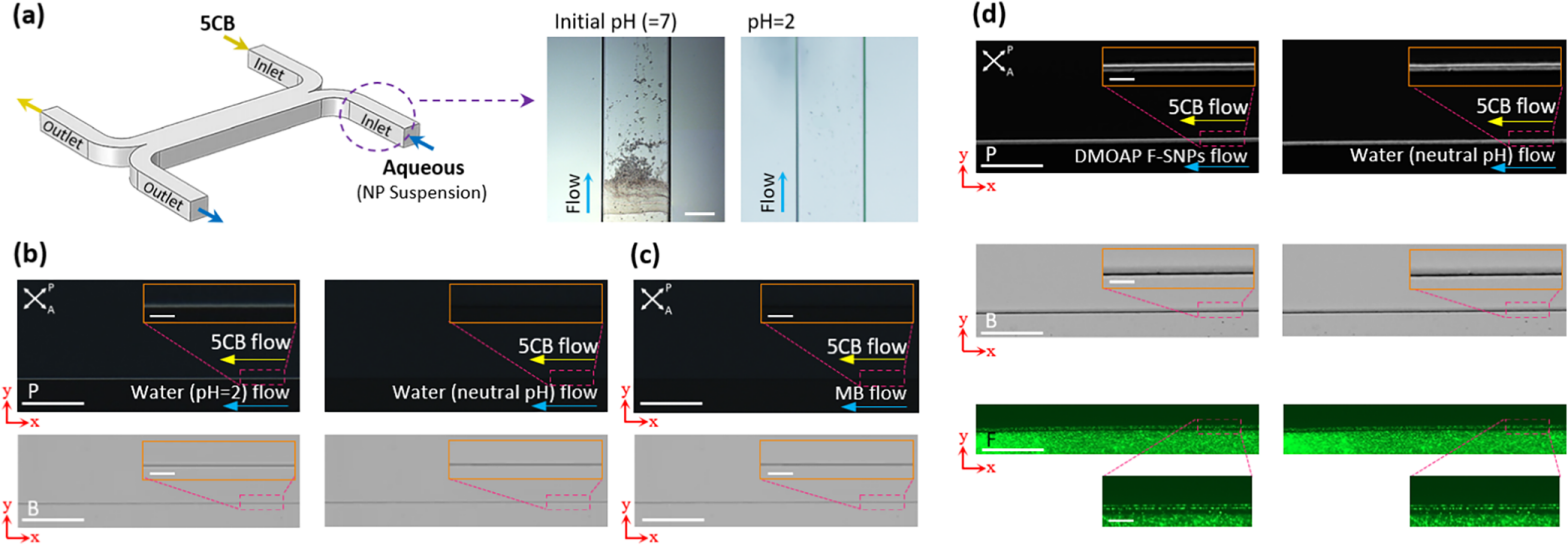
(a) Representative sketch of a microfluidic channel geometry. Using the nanoparticle suspension at its initial pH of approximately 7 blocks the channel inlet, owing to the adsorption of positively charged DMOAP F-SiNPs to the glass-PDMS walls. Meanwhile, nanoparticle suspension with pH = 2 effectively eliminated the blockage. (b) Temporary effect of water with pH = 2 on the LC anchoring at the 5CB–water interface, clarified by rinsing the channel with neutral pH water. (c) The introduction of aqueous MB flow into the microchannel confirmed that MB molecules do not induce a direct ordering transition at the LC-water interface. (d) Polarized (P), brightfield (B), and fluorescence (F) optical micrographs of the 5CB–aqueous DMOAP F-SiNPs with pH = 2 (∼10^9^ particles/mL) system, showing that the homeotropic state of the interface remained unchanged following the injection of water with neutral pH due to the effective adsorption of nanoparticles to the interface. Scale bars: (a–d) 100 and 10 μm for the corresponding zoomed sections.

In addition to the microfluidic experiments, we conducted experiments utilizing nanoparticle-assisted LC droplet sensors with an equivalent approach. Although microfluidic and LC droplet-based systems differ in geometry and interfacial physics, they are governed by the same underlying LC–interfacial interactions. Investigating both platforms provides complementary perspectives, enabling the decoupling of geometric effects from intrinsic interfacial responses. This integrated approach provides a deeper understanding of sensitivity and response dynamics, ultimately offering a more comprehensive and insightful evaluation of sensing performance.

As sketched in Figure 3a, 5CB droplet emulsions were prepared in suspensions of DMOAP F-SiNPs at pH = 2. After equilibration, specific concentrations of MB were added to the emulsion, and the steady-state configuration distributions of the 5CB droplets were subsequently investigated. Figure 3b depicts the brightfield, polarized, and fluorescence microscopy images of a representative 5CB droplet in the presence of ∼10^9^ particles/mL of DMOAP F-SiNPs. The observations indicated that a layer of nanoparticles was effectively adsorbed at the droplet interface, attributed to the physical interaction between negatively charged 5CB and positively charged DMOAP F-SiNPs (supported by ζ-potential measurements below). The locations of nanoparticles surrounding the interface of 5CB were shown by fluorescence confocal microscope (FCM) images obtained from the z-stack of the confocal micrographs (Figure 3c). Moreover, SEM images of polymerized 5CB droplets revealed the presence of a dense packing of nanoparticles at the droplet interface (Figure 3d). The magnified images confirmed that the nanoparticles fully covered the LC interface. It is important to note that the wrinkling observed in the full coverage of nanoparticles at the droplet interface resulted from the shrinkage of the polymerized droplets following the leaching of the unreacted mesogens. This phenomenon has been thoroughly documented in the literature.^41–45^

**Figure 3:**
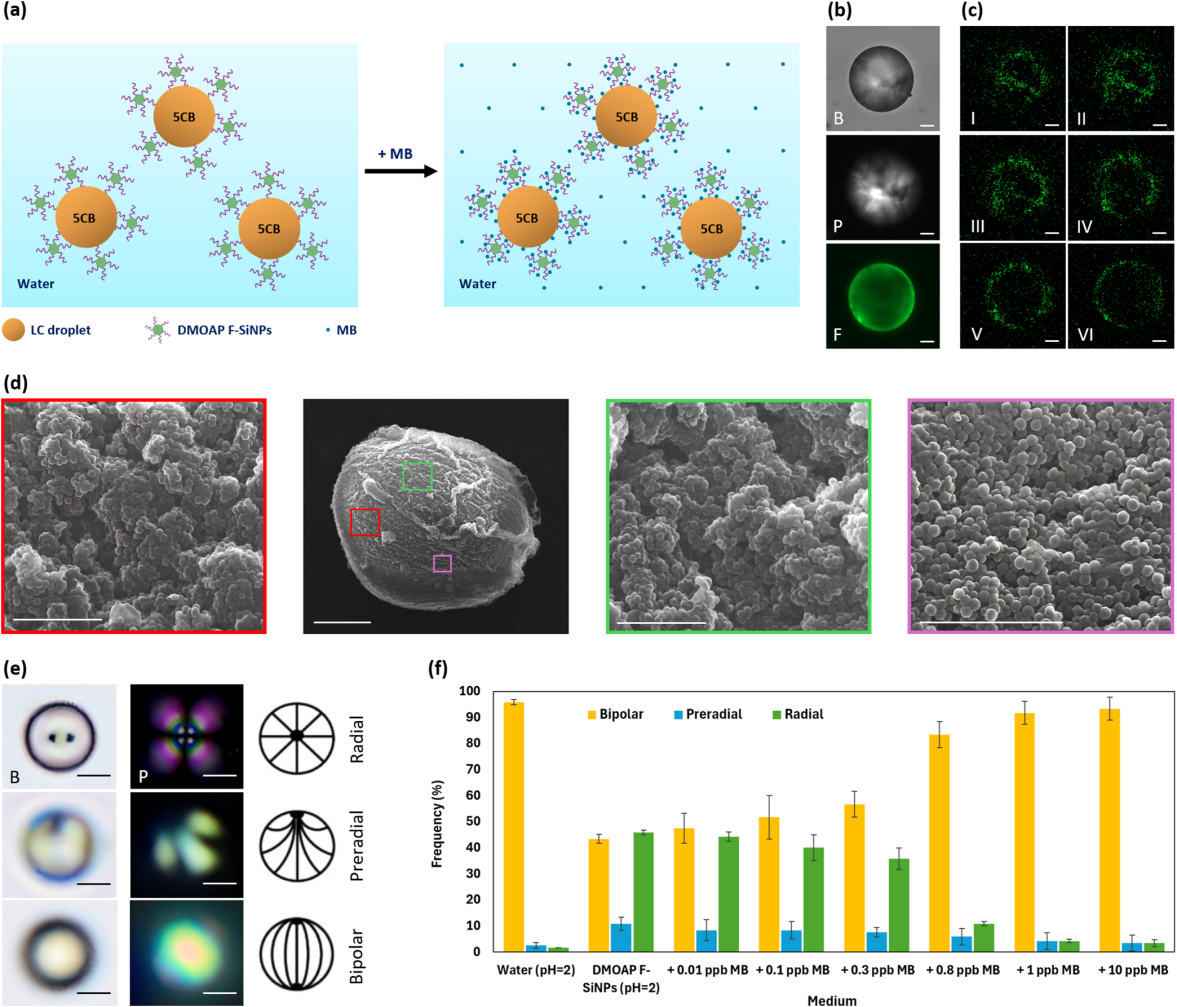
(a) Sketch of the experimental system of nanoparticle-decorated LC droplet sensors. (b) Brightfield (B), polarized (P), and fluorescence (F) microscopy images of a 5CB droplet in aqueous DMOAP F-SiNPs suspension with pH = 2 (∼10^9^ particles/mL). (c) FCM images collected from the z-stack micrographs indicate the locations of nanoparticles surrounding an LC droplet. I to VI show a gradual radial movement from the outer surface to the center of the droplet. (d) SEM images of the polymerized LC droplets after contacting with ∼10^9^ particles/mL DMOAP F-SiNPs suspension. The red, green, and purple squares represent the full coverage of the LC droplet with nanoparticles at different locations. (e) Brightfield and polarized light micrographs depict different 5C droplets corresponding to the bipolar, preradial, and radial director configurations as illustrated schematically. (f) Frequency of 5CB droplet configuration in aqueous DMOAP F-SiNPs suspension at pH = 2 (∼10^9^ particles/mL) with added MB of 0.01, 0.1, 0.3, 0.8, 1, and 10 ppb. The quantitative analyses were conducted through the evaluation of 60 to 90 droplets. Averages and error bars were collected from three independent measurements. Scale bars: (b, c) 20 μm, (d) 20 μm for the LC droplet and 1 μm for the magnified regions, (e) 5 μm.

The observed 5CB droplet configurations were categorized into three distinct groups, as exhibited in Figure 3e: *Bipolar* droplets, which arise from planar interfacial anchoring; *Preradial* droplets, encompassing a range of transitional states between bipolar and radial configurations (predominantly characterized by preradial configurations, along with a smaller number of escaped radial and axial droplets) originating from tilted interfacial anchoring; *Radial* droplets, which are formed due to the homeotropic interfacial anchoring of 5CB. As shown in Figure 3f, the initial 95.8 ± 1% bipolar configuration (1.7 ± 0.5% radial) of droplets in water with pH = 2 was observed to decrease to 43.3 ± 1.7% bipolar configuration (45.8 ± 0.85% radial) upon exposure to an acidic aqueous DMOAP F-SiNPs suspension. Adding MB at concentrations ranging from 0.01 to 10 ppb into the emulsion of 5CB within suspensions of DMOAP F-SiNPs at pH = 2 resulted in a response of droplets toward a progressive enhancement of bipolar configuration, accompanied by a corresponding reduction in radial configuration. Notably, the frequency of the bipolar configuration increased to 91.7 ± 4.4% and the radial configuration decreased to 4.2 ± 0.6%. The values exhibited relative stability at a concentration of 10 ppb. We reasoned that the response of the droplets may originate from the partitioning of analyte molecules at the interfaces, which subsequently restricts the interaction between the DMOAP molecules and the 5CB. This limitation diminishes the likelihood of perpendicular alignment of the 5CB at the interfaces. Such an intermolecular phenomenon is likely to lead to an increase in bipolar droplet configurations, as planar interfacial anchoring becomes more probable. We clarify that the emulsions were not prepared by integrating 5CB droplets into nanoparticle suspensions preequilibrated with aqueous solutions of MB at specified concentrations. Instead, we introduced various concentrations of MB into the initially prepared “Pickering” emulsions of 5CB droplets decorated with DMOAP F-SiNPs. Thus, the results of nanoparticle-decorated LC droplet sensors provided in this study are equivalent to the approach we followed in the microfluidic experiments.

To find the limit of detection (LOD) of MB for the nanoparticle-decorated LC-interfaced microfluidic sensors (prepared by ∼10^9^ particles/mL of DMOAP F-SiNPs), we investigated the response of 5CB at different concentrations of MB. Figure 4a indicates the representative response of the nanoparticle-decorated 5CB–aqueous interfaces to various MB concentrations. Accompanying sketches of the average LC director profiles are also provided to elucidate the underlying LC configurations in microfluidics, resulting in the shown optical appearances. Evidently, the nanoparticle-decorated 5CB interface remained unchanged following the injection of 0.1 ppb MB, while it exhibited a partial darkening upon the introduction of 0.3, 0.5, and 0.8 ppb of MB. The darkening process intensified with increasing MB concentration, attributed to the transition of additional regions from a homeotropic to a planar state, resulting from an enhanced adsorption of MB at the interface. Eventually, with the extinction of the homeotropic regions at the interface, the complete response occurred at 1 ppb MB.

**Figure 4:**
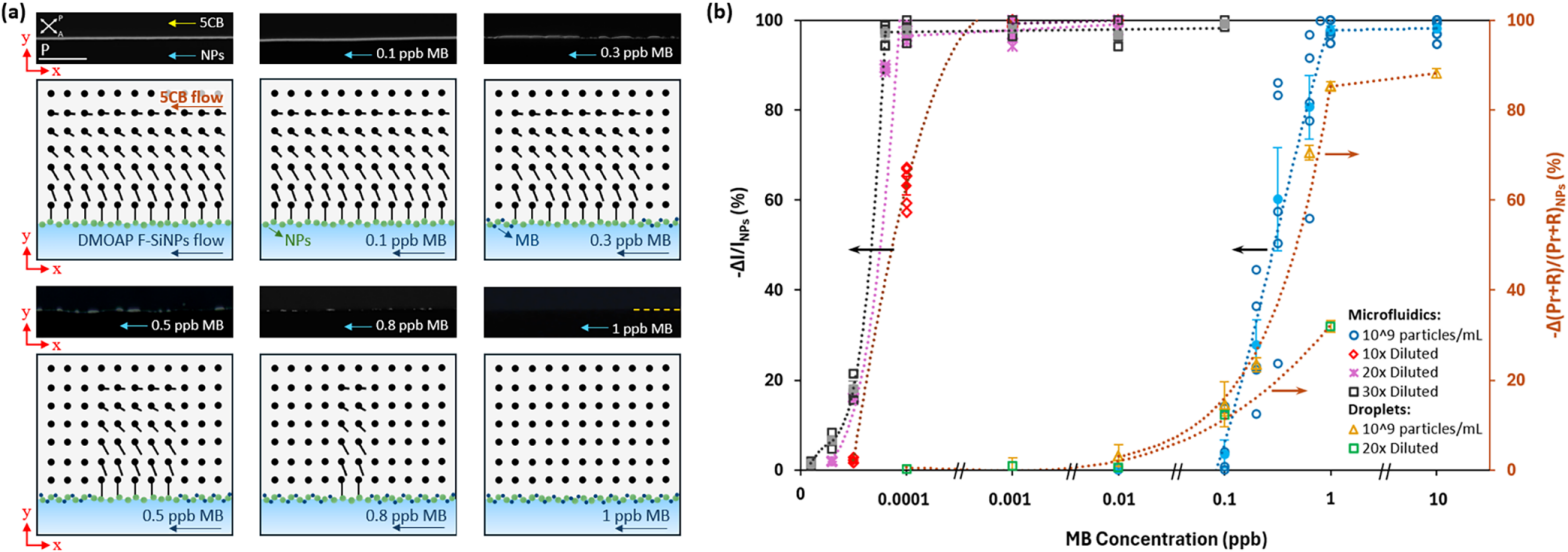
(a) Polarized (P) optical micrographs, along with the corresponding sketches, depicting the response of the nanoparticle-decorated LC flow sensor (∼10^9^ particles/mL of DMOAP F-SiNPs) to various MB concentrations. The dashed line in the optical micrograph shows the location of the LC-aqueous interface. (b) Response plot of nanoparticle-decorated LC sensors in microchannel (left axis) and droplet (right axis) systems to a range of MB concentrations from 0.01 ppt to 10 ppb, highlighting the LOD of the sensors for varying concentrations of DMOAP F-SiNPs. I, Pr, and R in y axes refer to intensity, preradial, and radial, respectively. Scale bars: 100 μm for the polarized micrographs.

To quantify responses from the microfluidic sensors, a signal formula derived from a generated function based on interfacial intensities (see Experimental) was utilized, demonstrating the MB concentration-dependent changes in the LC response. As highlighted in Figure 4b, the response of the microfluidic 5CB interface to the analyte reached its saturation at a concentration of 1 ppb and remained consistent beyond this threshold. In contrast, the average sensor response exhibited a gradual decline from 1 to 0.1 ppb, ultimately reaching less than 4% at a concentration of 0.1 ppb. Furthermore, no measurable response was detected at a concentration of 0.01 ppb. Accordingly, the LOD was found to be 0.1 ppb MB. Upon assessing the statistical analysis of the nanoparticle-assisted LC droplet sensors presented in Figure 3f, and applying a signal formula that accounts for the variations in the total number of preradial and radial droplets before and after adsorption of the analyte to the nanoparticle-decorated interface, it was revealed that the response curve for the droplet system utilizing the same ∼10^9^ particles/mL concentration of the nanoparticles exhibited a trend analogous to that observed in microfluidic sensor systems. These findings show that the LOD for the droplet system sensor was about 0.1 ppb, which aligns with the microfluidic results as shown in Figure 4b.

The results of the response experiments conducted in both microfluidic and droplet systems suggested that one potential response mechanism of the sensors stems from charge interactions occurring in two distinct stages. First, 5CB responds to the adsorption of the nanoparticles with a functionalized surface. Second, the nanoparticle-decorated 5CB interface responds to the adsorption of MB molecules. Utilizing ζ-potential measurements provided evidence on the underlying mechanism. As illustrated in Figure 5a, the ζ-potential of 5CB droplets was recorded as −49.3 ± 1.6 mV.^37,46–48^ The bare F-SiNPs exhibited a ζ-potential of −41.6 ± 1.2 mV. Following the functionalization of the nanoparticles with DMOAP, a ζ-potential of +30.7 ± 0.4 mV at pH = 2 was observed. These measurements were consistent with the past data.^37^ The high positive ζ-potential value encouraged the adsorption of the DMOAP-functionalized nanoparticles onto the negatively charged interfaces of 5CB. This phenomenon was evidenced by the ζ-potential of 5CB droplets emulsion prepared in an aqueous DMOAP F-SiNPs suspension at pH = 2, which was measured to be −1.7 ± 0.4 mV. The findings indicated that the adsorption of nanoparticles led to a significant reduction in the negative charge at the interfaces of 5CB, ultimately bringing the overall ζ-potential close to neutrality. Consequently, there exists the potential for MB molecules to adsorb onto the nanoparticle-decorated 5CB–aqueous interface. This adsorption process is likely to occur through various mechanisms, which will be elaborated upon in the subsequent discussion. Among these mechanisms, electrostatic interactions represent one of the plausible contributing factors. The SEM images presented in Figure 5b demonstrated that the density of the adsorbed nanoparticles at the interfaces of the 5CB droplets was significantly decreased when droplets were exposed to a less concentrated nanoparticle suspension. Specifically, in contrast to the full coverage of the droplet interfaces using ∼10^9^ particles/mL of DMOAP F-SiNPs (as illustrated in Figure 3d), exposure of 5CB droplets to 20× diluted nanoparticle suspension led to partial interfacial coverage. This observation prompted a critical question regarding the implications of partial interfacial coverage. To explore this further, we employed a 60× diluted aqueous DMOAP F-SiNPs suspension in the ζ-potential measurements investigations. This dilution was adopted to ascertain the ζ-potential of complex emulsions containing 5CB droplets, DMOAP F-SiNPs, and MB, as the use of diluted nanoparticle suspensions facilitated more accurate and reliable measurements. Figure 5a demonstrates that introducing 0.01 ppt MB to the emulsion of 5CB droplets prepared in an aqueous suspension of DMOAP F-SiNPs at pH = 2 resulted in a shift in the ζ-potential toward a marginally positive value of 0.5 ± 0.2 mV. Upon increasing the MB concentration, a slight enhancement in the ζ-potential was observed, reaching +16.9 ± 2.5 mV at a concentration of 0.1 ppb MB. Control experiments also showed that adding 0.1 ppb MB to the nanoparticle suspensions did not change the ζ-potential of DMOAP F-SiNPs due to electrostatic repulsion between the positively charged nanoparticles and MB molecules. Thus, we reasoned that the complex charging of the oil–water interfaces was critical in facilitating the interactions of the analytical species with the adsorbed nanoparticles at 5CB interfaces. In light of these findings, we sought to explore the detection of trace-level concentrations of MB utilizing nanoparticle-decorated LC sensors in both droplet systems and microchannels.

**Figure 5:**
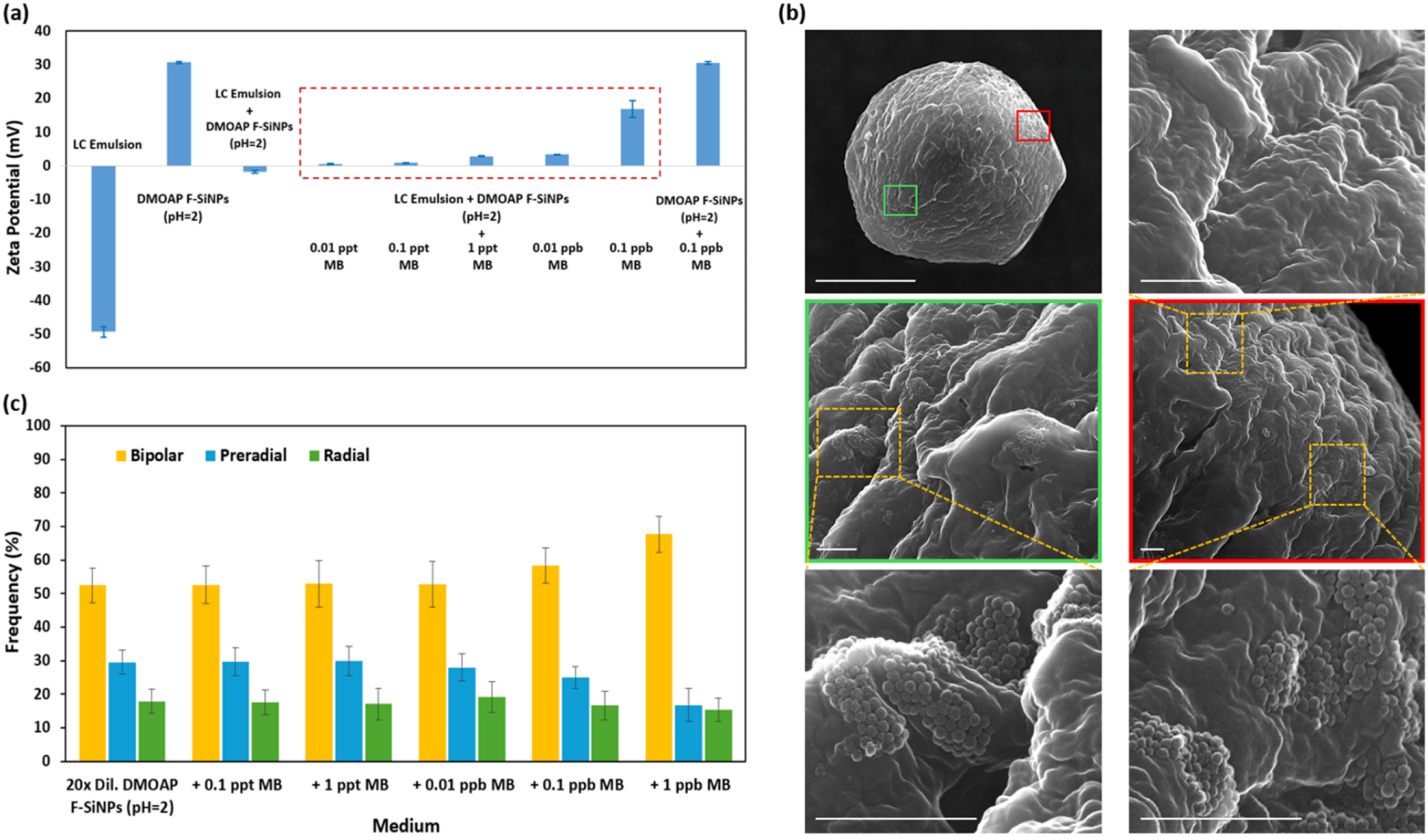
(a) Variation of ζ-potential for the systems involving 5CB-in-water emulsion, 60× diluted aqueous DMOAP F-SiNPs suspension with pH = 2, and various concentrations of MB. (b) SEM images of the polymerized LC droplets after contacting with 20× diluted DMOAP F-SiNPs suspension. The partial coverage of LC droplets, including areas with adsorbed nanoparticles as well as regions devoid of them, is illustrated in two distinct zones identified by red and green squares. (c) Configuration distribution of Pickering emulsion of 5CB droplet in aqueous 20× diluted DMOAP F-SiNPs suspension at pH = 2 with added MB of 0.0001, 0.001, 0.01, 0.1, and 1 ppb. Scale bars: (b) 20 μm for the LC droplet and 1 μm for the magnified region.

Nanoparticles formed a dense interface-adsorbed phase, enforcing dominant anchoring across the entire surface. For homeotropic-inducing nanoparticles, this shifts the droplet predominantly to a radial configuration (as confirmed by the frequency of droplet configurations in Figure 3f). However, partial coverage resulted in a heterogeneous or “patchy” 5CB–aqueous interface. The presence of the nanoparticles created localized areas with altered interfacial energy and interfacial anchoring conditions, while the remaining nanoparticle-lean interface retained its original anchoring. This mismatch created localized elastic distortions in the nematic director field, manifesting as topological defects that served as energetic traps for nearby nanoparticles. As a result, nanoparticles migrated to regions of high elastic stress and formed aggregates.^49,50^ These aggregates would subsequently lead to preradial configurations as a consequence of defect pinning.^44^ The phenomenon elucidated the significant increase in the frequency of preradial configurations relative to radial configurations in a Pickering emulsion composed of 5CB droplets within a 20× diluted aqueous nanoparticle suspension (Figure 5c). In the vicinity of defect regions, LC mesogens were susceptible to small perturbations because the director field was already distorted and energetically penalized. When an analyte adsorbs to the nanoparticles, it slightly alters the local anchoring strength or orientation. This minor local change propagates through the elastic LC matrix, resulting in a noticeable realignment that can amplify sensing signals. Pickering emulsions of 5CB droplets decorated with 20× diluted DMOAP F-SiNPs exhibited a response to analyte concentrations as low as 0.1 ppb (Figure 5c). However, through our investigations using microfluidics, we uncovered something truly noteworthy about continuous interfaces in terms of sensitivity, which we will elaborate on below.

To examine the response of the nanoparticle-decorated microfluidic LC-aqueous interfaces to lower concentrations of analyte in the aqueous medium (≤0.1 ppb, which is the LOD determined for ∼10^9^ particles/mL of DMOAP F-SiNPs), the 5CB–aqueous interface was decorated using 10× diluted DMOAP F-SiNPs, followed by the injection of a 0.1 ppb MB aqueous solution into the channel. These experiments were performed at low bulk and interfacial shear stress with equal inlet pressures to avoid high shear stress effects, aligning with prior experiments. The incubation of the channel using a 10× diluted nanoparticle suspension led to the formation of a thinner, bright appearance at the 5CB–water interface, compared to observations made with the concentrated nanosuspension. The polarized micrographs in Figure 6a indicate that the bright appearance of the 5CB–water interface progressively darkened over time as MB molecules adsorbed onto the nanoparticles, shifting to planar anchoring. The brightfield microscopy images also reflected this transition. The sensing process was completed in 25 min for a 0.1 ppb concentration of MB. In contrast to the fluorescence micrographs obtained from channels incubated with nondiluted nanosuspension, which revealed a substantial number of nanoparticles adsorbed to the 5CB–water interface as well as to the glass and PDMS surfaces in the bulk of aqueous media, the current experiments demonstrate a markedly reduced quantity of adsorbed nanoparticles. The interaction between MB molecules and these adsorbed nanoparticles was responsible for the anchoring transition of 5CB to the planar state. At the end of the sensing process, rinsing the channel with fresh water provided evidence of the reversibility of the system (Figure 6b). During this process, the nanoparticles remained positioned at the interface, while the dye molecules previously adsorbed onto the nanoparticle-decorated 5CB interface were effectively desorbed and carried away by the flowing water. Figure 6c indicates the ultimate response of a 10× diluted nanoparticle-decorated LC flow sensor to various trace-level concentrations of MB. Corresponding sketches are included to clarify the mechanism that drives the responses. The thin, bright layer arose from the interfacial homeotropic/tilted alignment of 5CB, resulting from the partial coverage of nanoparticles at the interface along the glass side (to be discussed later). The nanoparticle-decorated 5CB–aqueous interface showed no change with the introduction of 0.05 ppt of MB. However, a partial darkening was observed after the injection of 0.1 ppt of MB. The increased adsorption of MB molecules at the interface resulted in a transition to a planar anchoring, achieving a complete response at a concentration of 1 ppt of MB.

**Figure 6:**
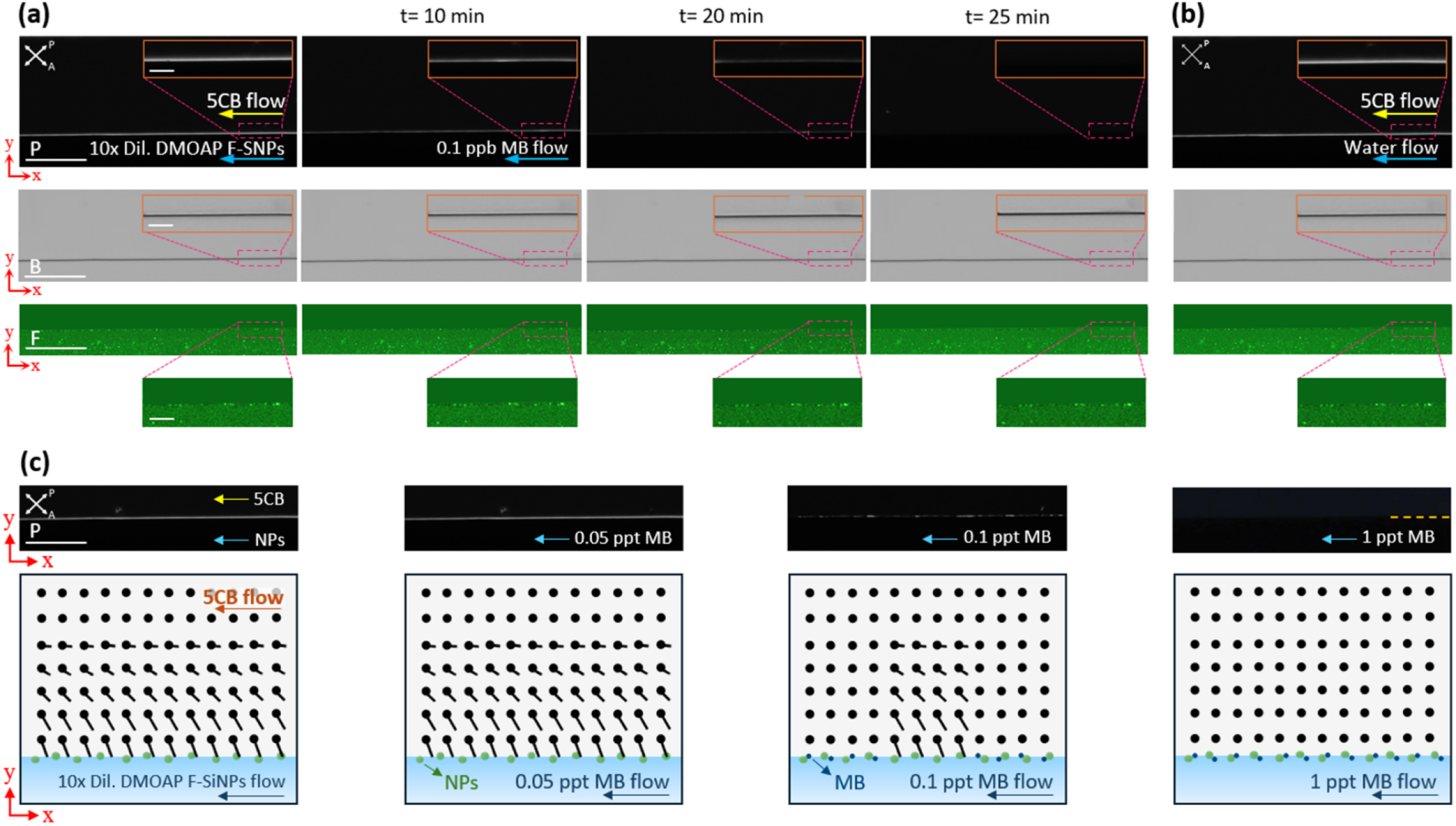
(a) Response of the nanoparticle-decorated LC flow sensor (10× diluted DMOAP F-SiNPs) to 0.1 ppb MB concentration over time, showing the orientational ordering transition of the interface from homeotropic/tilted to planar anchoring upon adsorption of MB molecules to the interface in 25 min. (b) Washing the channel with water after the sensing process shows the reversibility of the system, enabling the interface to revert to the homeotropic/tilted state upon analyte elimination. (c) Polarized optical micrographs, accompanied by the corresponding sketches, illustrating the definitive response of the 10× diluted nanoparticle-decorated LC flow sensor to various ultralow MB concentrations. Scale bars: 100 μm for the polarized (P), brightfield (B), and fluorescence (F) micrographs and 10 μm for their zoomed parts.

As presented in Figure 4b, a decrease in the MB concentration from 0.1 ppb to 1 ppt resulted in the sensor maintaining a robust response to the analyte. Subsequent reductions in the MB concentration led to a decline in the average sensor response, ultimately diminishing to less than 3% at a concentration of 0.05 ppt. Therefore, a 10-fold dilution of nanoparticles (partial interfacial coverage) during the microfluidic experiments achieved a substantial decrease in the LOD by 4 orders of magnitude. To further investigate the assessment of nanoparticle-decorated LC flow sensors in response to ultralow concentrations of MB, we utilized nanoparticles diluted to 20× and 30× within the sensors. It was observed that in the case of 10× diluted nanoparticles, the average response of 5CB to MB decreased to approximately 60% at a concentration of 0.1 ppt of the analyte. Conversely, both the 20× and 30× diluted nanoparticles sustained their maximum response values at the same concentration. Furthermore, the LODs of sensors utilizing 20× and 30× diluted nanoparticles decreased to 0.03 and 0.01 ppt of MB, respectively. These results were found to be closely aligned with each other (Figure 4b). Consequently, further dilution of the nanosuspension is unlikely to yield a significant outcome. This observation was confirmed through experiments conducted with up to 100× diluted nanosuspensions.

In essence, we showed that in terms of achieving highly sensitive nanoparticle-integrated LC-based sensors, soft interfaced continuous systems in microfluidic platforms act as a wizard that facilitates the detection of analytes at trace-level concentrations in water, featuring sharp changes in the easily observable optical output. At this stage, the fundamental questions that emerge are

(i) *What are the underlying mechanisms that explain how nanoparticle-decorated LC flow sensors respond when subjected to both concentrated and diluted nanoparticle solutions?*.
(ii) *What is the selectivity profile of the developed sensor when it is exposed to diverse analytes in an aqueous environment?*

To comprehensively address these inquiries, we undertook a series of supporting experiments, the findings of which are detailed along with the discussion of the critical role of the nanoparticle-decorated interfaces of the microfluidic LC systems.

The remarkable enhancement in sensor performance can be fundamentally linked to the structures of the LC–aqueous interface, specifically to the localization of the nanoparticles at the interface. Using fluorescence confocal polarizing microscope (FCPM) imaging collected at the midplanes of the channels, we were able to conduct a thorough investigation of the 5CB–aqueous phase interface in the absence and presence of nanoparticles at different concentrations, as well as the response of the nanoparticle-decorated 5CB–aqueous interface flow sensor to the analyte. The polarized, brightfield, and FCPM micrographs exhibited in column I of Figure 7 illustrated the planar anchoring of 5CB at the interface. In addition, z-stack cross-sectional FCPM images collected at 0° and 90° polarization of the fluorescence excitation light revealed that the interface was not flat and displayed a minor, slightly curved inclination. Upon the decoration of the interface with suspensions of ∼10^9^ particles/mL DMOAP F-SiNPs, a thick, bright layer was formed at the interface, as depicted in the polarized, brightfield, and FCPM images presented in column II of Figure 7. This thick layer indicated the homeotropic anchoring of 5CB, resulting from the considerable adsorption of nanoparticles at the interface. The presence of the bright layer was also evident in the z-stack FCPM image obtained at 90° polarization of the fluorescence excitation light (shown with a yellow arrow in the micrograph). The three-Dimensional (3D) view of the 5CB–aqueous interface obtained from z-stack FCM imaging supports the formation of a layer of nanoparticles at the inclined interface. The distribution of nanoparticles was effectively observed between the glass and PDMS; however, the adsorption on the glass side was more pronounced, where aggregations of nanoparticles also formed. We associated this with several factors, including the interaction of silanol groups with a higher density on the glass surface. Additionally, gravity plays a role due to the aggregation of the nanoparticles, as well as the strain on the LC side that would facilitate particle motion toward the glass interface, induced by elastic energy resulting from anchoring. The thick, bright appearance of the 5CB–aqueous interface completely faded following the adsorption of MB molecules onto the nanoparticles at the interface, as shown in column III of Figure 7. The 3D structure of the 5CB–aqueous interface obtained from z-stack FCM imaging indicated the presence of nanoparticles at the interface after the MB injection. The layer of nanoparticles was observed to be less intense compared to the one depicted in the 3D image in column II. This can be attributed to the minor replacement of nanoparticles, which occurred as a result of disturbing the system during the exchange of the nanoparticle suspension and analyte solution inlets. However, this does not explain the observed response, as the quantity of adsorbed nanoparticles remained quite sufficient to induce homeotropic interfacial anchoring at the 5CB–aqueous interface.

**Figure 7:**
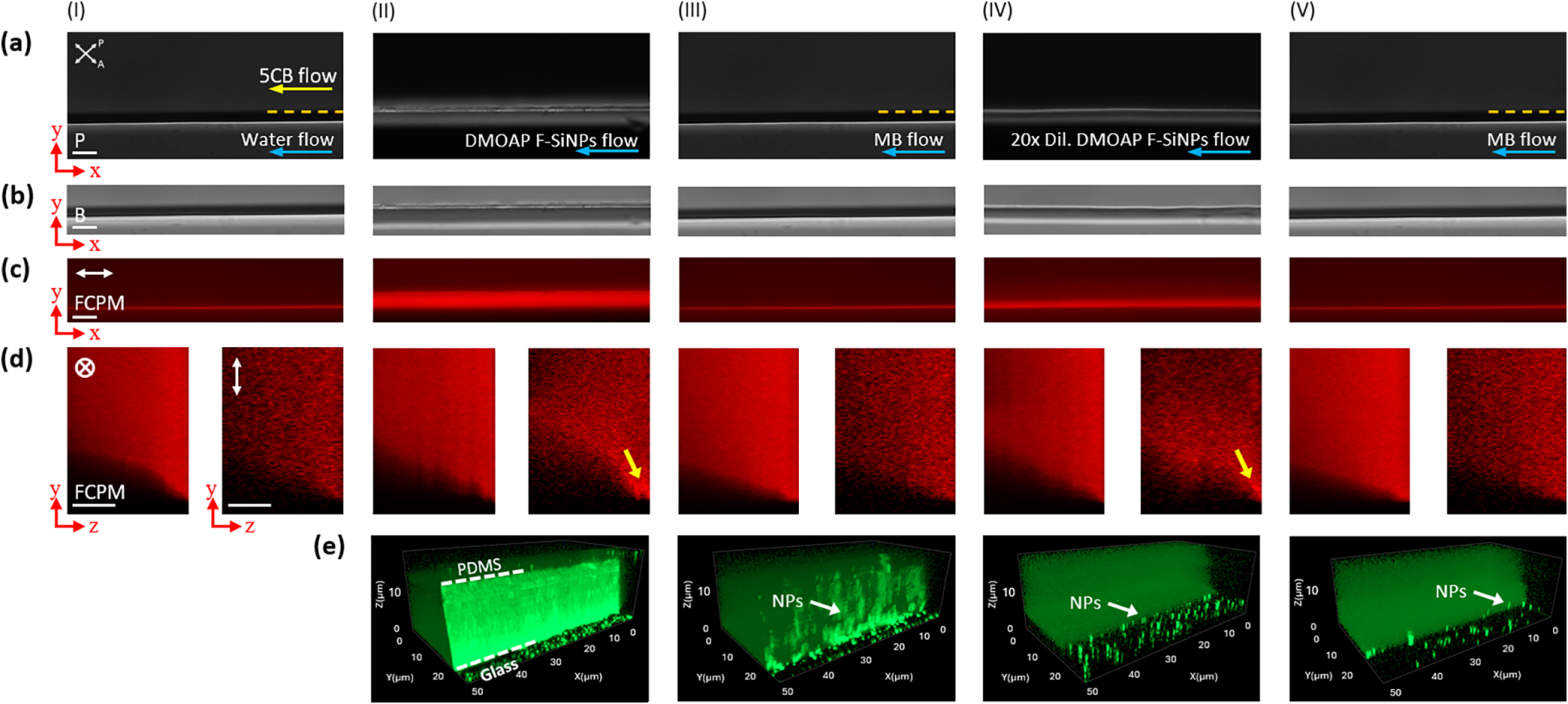
(a) Polarized (P), (b) brightfield (B), and (c) fluorescence confocal polarizing microscope (FCPM) images (taken from midplanes) of five systems including the (I) 5CB–water, (II) 5CB–aqueous DMOAP F-SiNPs with pH = 2 (∼10^9^ particles/mL), (III) the nanoparticle-decorated LC flow sensor (∼10^9^ particles/mL of DMOAP F-SiNPs) responding to 1 ppb MB concentration, (IV) 5CB–aqueous 20× diluted DMOAP F-SiNPs with pH = 2, and (V) the nanoparticle-decorated LC flow sensor (20× diluted DMOAP F-SiNPs) responding to 0.1 ppt MB concentration. The images show the transition of orientational ordering at the 5CB–water interface from planar to homeotropic/tilted anchoring with concentrated and diluted nanoparticles, respectively, and shifting to planar after the nanoparticle-decorated LC flow sensors respond to MB. (d) Z-stack cross-sectional FCPM images of 5CB (with 0.01% Nile red fluorophore) at the aqueous interface (found to be inclined) acquired at 0° and 90° polarization of the fluorescence excitation light. (e) 3D structure of the 5CB–aqueous interface obtained from z-stack FCM imaging, demonstrating the distribution of nanoparticles at the inclined interface. Scale bars: (a, b, c) 10 μm, (d) 5 μm.

To comprehend the adsorption of nanoparticles at the interface and analyze the subsequent response of the nanoparticle-decorated LC flow sensor in relation to the quantity of MB molecules, we conducted a numerical evaluation. Considering the effective 5CB–aqueous interface as a flat plane measuring 1.5 mm in length and 14 μm in width (depth of the microchannels), we calculated that 5 × 10^6^ particles with an average size of 70 nm can accommodate the interface to establish a close-packed monolayer of nanoparticles. A total of 50 μL of the aqueous DMOAP F-SiNPs suspension, with a concentration of ∼10^9^ particles/mL, was employed to coat the interface. Consequently, this resulted in the presence of 5 × 10^7^ particles within the microchannel, which was adequate for the formation of a monolayer. Excessive nanoparticles may accumulate at the interface, particularly along the glass side, while some adsorb to the glass-PDMS walls or are expelled from the outlet. Recently, we have shown that nanoparticle-assisted LC droplet sensors respond to the sub-ppb-level concentrations of MB in aqueous medium, which corresponds to an interfacial coverage of around 20% of a monolayer at the interface of nanoparticles.^31^ Accordingly, the total number of MB molecules required to cover 20% of the interfacial area of the adsorbed monolayer of nanoparticles was 1.5 × 10^10^ molecules. About 50 μL of 1 ppb MB, the threshold concentration that yielded a complete sensor response to the analyte, was consumed during the detection experiments. Hence, there were 9.4 × 10^10^ molecules of MB within the microchannel, which were effectively able to interact with the nanoparticles at the 5CB–aqueous interface. This interaction induced a transition in the interfacial orientational ordering of 5CB from homeotropic to planar anchoring.

The polarized, brightfield, and FCPM micrographs illustrated in column IV of Figure 7 showed that decorating the interface using a 20× dilution of aqueous ∼10^9^ particles/mL of DMOAP F-SiNPs suspension led to the formation of a thin, bright layer at the interface. The z-stack FCPM image obtained at 90° polarization of the fluorescence excitation light also reflected the presence of this bright layer (shown with a yellow arrow in the micrograph). The observed thinner bright layer (compared to column II) originated from the localized director field straining of 5CB, caused by the localized adsorption of nanoparticles at the interface along the glass side, as evidenced by the 3D presentation of the z-stack FCM imaging conducted at the 5CB–aqueous interface. Upon the adsorption of MB onto the nanoparticles at the interface, as illustrated in column V of Figure 7, the bright appearance of the 5CB–aqueous interface entirely vanished. The 3D structure of the z-stack FCM image confirmed the presence of nanoparticles at the interface during the response to the analyte, where MB molecules interacted with the nanoparticles, resulting in an ordering transition of 5CB to planar anchoring. The substantial decrease in the number of nanoparticles notably restricts the formation of a monolayer within the microchannel. Specifically, in 50 μL of 20× diluted nanoparticle suspensions, the total quantity of available nanoparticles corresponds to one close-packed monolayer of nanoparticles at the interface. A considerable amount of these particles was lost through the channel due to adherence to the glass walls or expulsion from the outlet. The remaining nanoparticles tend to preferentially adsorb to the interface along the glass side. The 3D visualization of the z-stack FCM image displayed in column IV of Figure 7 verifies that the adsorption of nanoparticles to the glass side of the interface was minimal. Thus, 9.4 × 10^6^ molecules of MB, equivalent to 50 μL of 0.1 ppt MB—the threshold analyte concentration for complete sensor response—was sufficient for interaction with the nanoparticles at the 5CB–aqueous interface. This interaction caused a shift in the orientational ordering of the LC from homeotropic/tilted to planar anchoring.

Based on the structural characterizations carried out by microscopy, we sketched the proposed LC director configurations, as depicted in Figure 8, which also summarizes the response mechanism of the nanoparticle-decorated LC flow sensors. The planar anchoring of 5CB at the interface of pure water was shown earlier in Figure 1c. The interface was found to maintain an inclination with a slight curvature through FCPM images. Figure 8a demonstrates the perpendicular alignment of 5CB at the interface after decoration of the interface with a concentrated nanosuspension. The analysis of the x–z and y–z views of the z-stack FCM micrographs revealed that the resulting homeotropic anchoring was ascribed to the full coverage of the interface with nanoparticles. This full coverage, along with the inclination of the interface, substantiated the observation of a double line of nanoparticles in sensing experiments (Figures 1e,g,h and 2d) when examining the microchannels from the top (x–y view). Specifically, the lower and upper lines correspond to the adsorbed nanoparticle aggregates at the glass and the PDMS contact lines of the interface, respectively. Following the adsorption of MB molecules to the interface in the presence of nanoparticles, the orientational ordering of the 5CB–aqueous phase shifted from homeotropic to planar anchoring (Figure 8b). Treating the microchannel with 20× diluted nanoparticle suspensions resulted in the localization of interface-adsorbed nanoparticles along the glass-contact line, as shown in Figure 8c. This phenomenon explained the observation of a single line of nanoparticles in the sensing experiments (Figure 6a,b), in contrast to the appearance observed with nondiluted nanoparticles. The contact line deposition of the nanoparticles is essential for achieving enhanced sensitivity at low concentrations within microfluidic channels. This significance is underscored by the results from equivalent droplet experiments (Figure 5c), which did not demonstrate a reduction in the LOD. Accordingly, LC ordering was locally strained to possess bend distortions as shown in Figure 8c. Upon the adsorption of MB onto the nanoparticles at the interface, the interfacial anchoring of LC switched to a planar state as sketched in Figure 8d. When nanoparticles adsorb at the interface, regions exhibiting bent distortions ranging from 0 to 90° extend several micrometers in length. In the case of a concentrated nanoparticle-decorated interface, the strained interfacial LC region extended up to significant distances away from the aqueous interface, indicating a considerable storage of elastic energy in proximity to the aqueous interface. In contrast, for the diluted nanoparticle-decorated interface, the elastic energy density due to straining was greater compared to the concentrated nanoparticle-decorated interface. Nayani et al. documented a comparable procedure in an LC-based sensor comprised of a synthesized nitrile-containing LC mixture that exhibited planar anchoring at a free surface to air while maintaining a homeotropic orientation at a solid surface decorated with transition metal cations. They demonstrated that the planar orientation of the LC at the free surface made possible the design of an initially strained state of the LC, thus allowing the elastic energy stored in the initial state of the LC to be released during the response to a targeted analyte, leading to the attainment of a strain-free state.^51^

**Figure 8:**
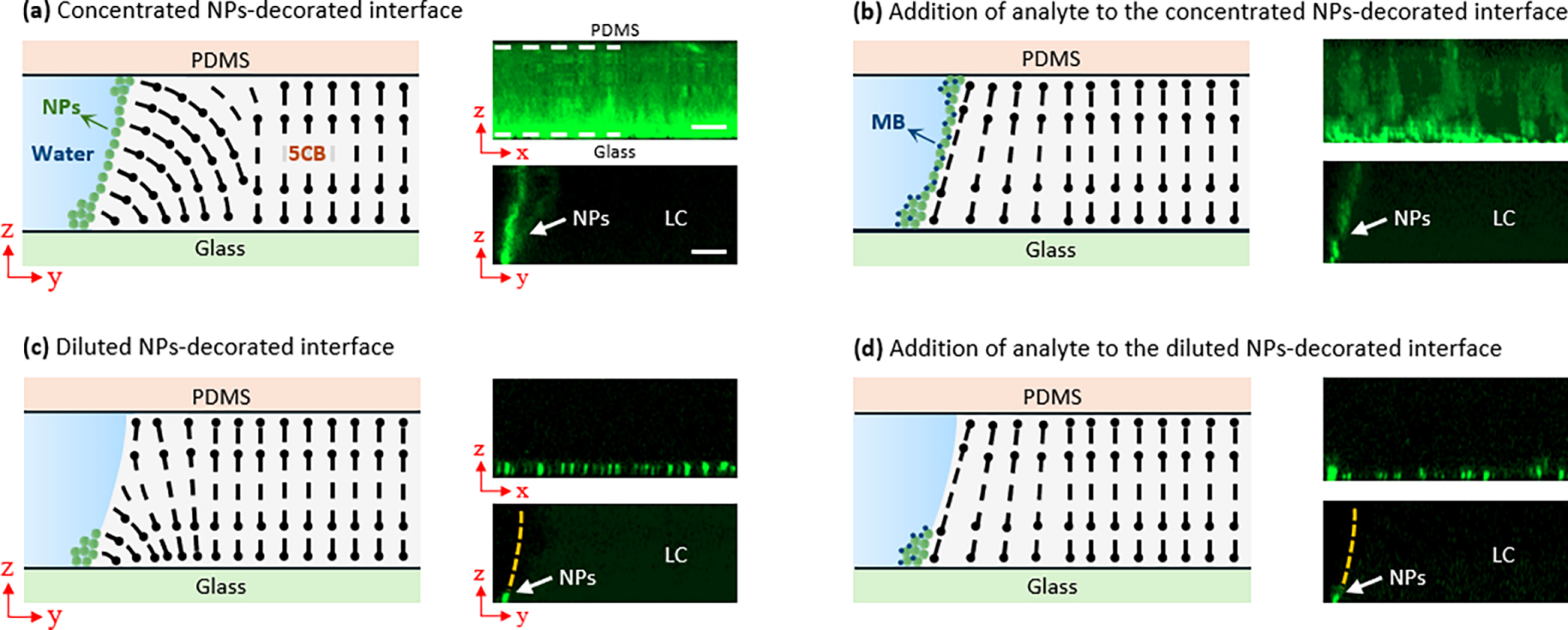
Representative sketches of the LC director profiles under weak flow conditions, which are delineated based on the distribution of nanoparticles at the interface, as derived from z-stack FCM micrographs (x–z and y–z views) for (a) 5CB–aqueous DMOAP F-SiNPs with pH = 2 (∼10^9^ particles/mL), (b) the nanoparticle-decorated LC flow sensor (∼10^9^ particles/mL of DMOAP F-SiNPs) responding to 1 ppb MB concentration, (c) 5CB–aqueous 20× diluted DMOAP F-SiNPs with pH = 2, and (d) the nanoparticle-decorated LC flow sensor (20× diluted DMOAP F-SiNPs) responding to 0.1 ppt MB concentration. Scale bars: 5 μm for z-stack micrographs.

To elaborate, the free energy density associated with the LC strain can be estimated by the so-called Frank–Oseen equation. One elastic constant approximation of the Frank–Oseen equation is expressed as follows^9^

1 $$F_{\mathrm{elastic}}=\frac{1}{2}{K\mathrm{\theta ̇}}^{2}$$

where K and θ̇ are the elastic constant and the spatial gradient of the director orientation angle, respectively. For 5CB, K is typically 10^–11^ N,^52^ and for a homeotropic state exhibiting a 90° bend, θ̇ is equal to $\frac{\pi /2}{L}$ , where *L* is the characteristic length of the bend distortion.

The analysis of the concentrated nanoparticle-decorated interface revealed that L was 14 μm, reflecting an even distribution of nanoparticles along the channel depth, with a resultant value of 8.8 × 10^–7^ J/m^2^ for the elastic energy of deformation per unit area. Conversely, the L of the bend distortion observed at the diluted nanoparticles-decorated interface was determined as ∼1 μm, with respect to the localized adsorption along the glass side as shown in Figure 8c, resulting in an *F*_elastic_ of 1.2 × 10^–5^ J/m^2^. On the other hand, the typical values of interfacial anchoring energy (W) for strong anchoring range in the order of 10^–5^ – 10^–4^ J/m^2^.^53,54^ Accordingly, for the concentrated nanoparticle-decorated interface, W ≫ *F*_elastic_. This indicates two key points: First, the 90° bend distortions occur entirely at the interface with homeotropic anchoring owing to the monolayer adsorption of nanoparticles at the 5CB–aqueous interface. Second, this anchoring is determined predominantly by interfacial interactions after analyte introduction. In contrast, for the diluted nanoparticles-decorated interface, W is comparable to *F*_elastic_. As a result, a true 90° bend distortions hardly occur due to the elastic energy penalty, indicating that the presence of several localized nanoparticles at the glass interface primarily results in tilted anchoring, as evidenced by low birefringence at the interface (Figure 6a). Besides, the anchoring to the alignment of 5CB at the nanoparticles interface becomes increasingly sensitive to minor variations in the chemistries of the nanoparticle-LC interface, which can be triggered by changes in chemical interactions, resulting in significantly amplified responses.

The sensor response argument can also be correlated to Figure 4b, which illustrates that the results for Pickering emulsions of 5CB droplets decorated with concentrated nanoparticles closely align with those observed in microfluidic sensor systems. This consistency arises because the interface in both systems is primarily influenced by the interfacial anchoring energy rather than by the elastic energy of deformation. In contrast, for interfaces decorated with diluted nanoparticles, the findings for the Pickering emulsions of 5CB droplets diverge significantly from those of the microfluidic-interfaced systems. Specifically, the microfluidic systems adhere to a more critical competition between *F*_elastic_ and W, which is expected to be the reason why LOD was enhanced by 4 orders of magnitude. However, the droplet systems exhibited much less sensitivity in their responses, as the nanoparticles at the droplet interface do not induce the same magnitude of strain. As previously mentioned, these nanoparticles form patches on the droplets, resulting in a predominantly preradial configuration that does not generate a significant strain within the droplets.

To address the selectivity of the sensor, we conducted comprehensive experiments with a range of analytes, including dyes, micropollutants (such as pharmaceuticals and personal care products (PPCPs) and industrial chemicals), a protein, and amino acids, was analyzed through these experiments (Table 1).^57–65^ Among the employed model analytes, the 20× diluted nanoparticle-decorated LC flow sensors responded to concentrations ≥ 0.1 ppt of MB, Acridine Orange, Nile Red, Diclofenac, Indomethacin sodium hydrate, and perfluorooctanoic acid (PFOA). As a plausible mechanism, we have already demonstrated through ζ-potential measurements that the analyte adsorbs onto the nanoparticle-decorated LC interface via electrostatic interactions. However, a thorough analysis of the characteristics of these molecules provided further insights into the potential interactions between the responding analytes and the nanoparticle-decorated LC interface. We found that hydrophobic interactions and π-π stacking can also play roles in the response of the sensors to these analytes. We noticed that each of these analytes possesses hydrophobic regions, including aromatic rings within their chemical structures (except PFOA, which contains a perfluorinated hydrophobic region). Specifically, MB and Acridine Orange have three fused rings, Nile Red possesses four fused rings, Diclofenac consists of dichlorophenyl and phenylacetic acid groups, and Indomethacin features an indole core with *p*-chlorobenzoyl substituent. The aromatic groups of all five compounds can interact with hydrophobic biphenyl and pentyl chains of 5CB, and can also engage in π-π stacking with the biphenyl system of 5CB molecules at the nanoparticle interface.

**Table 1:**
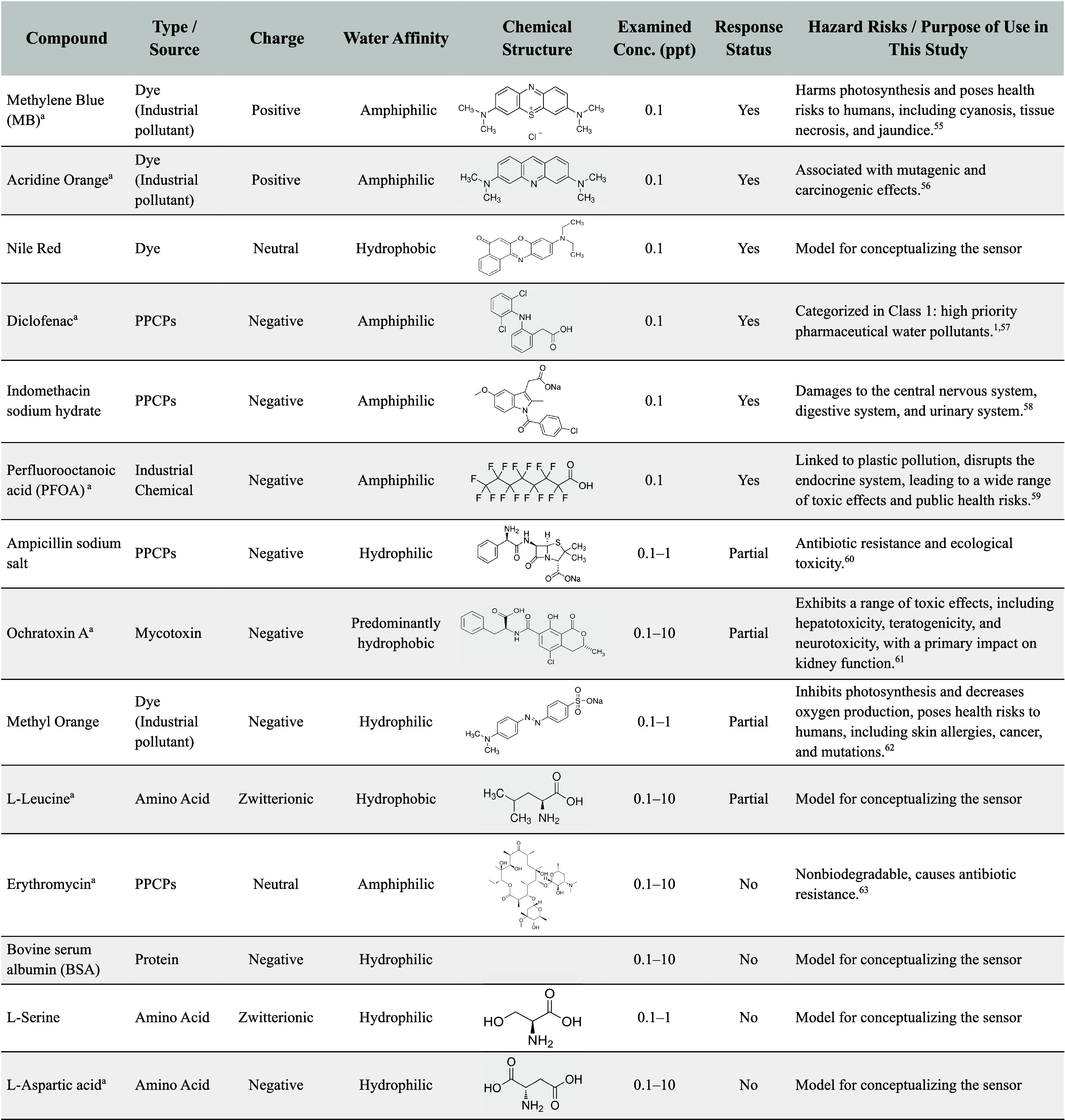
Selectivity of 20× Diluted Nanoparticle-Decorated LC Flow Sensors toward the Detection of Various Analytes

a The response experiments for these analytes were conducted at least three times.

Further, the sensor exhibited partial responses to concentrations of 0.1–10 ppt for Ampicillin sodium salt, Ochratoxin A, Methyl Orange, and l-Leucine. These compounds also contain hydrophobic aromatic rings within their chemical structures (except l-Leucine, which possesses a hydrophobic aliphatic chain). In detail, Ampicillin sodium salt has a phenyl ring, Ochratoxin A consists of a chlorinated phenyl ring and an isocoumarin ring system, and Methyl Orange includes two benzene rings. Similarly, the partial response of the sensor at this concentration to these analytes may originate from electrostatic and hydrophobic interactions, as well as π-π stacking between the analytes containing aromatics and the 5CB. Moreover, it was observed that the sensor did not respond to analytes such as erythromycin, BSA, l-serine, and l-aspartic acid. These compounds either have no aromatic groups (or limited accessible aromatics for BSA) in their chemical structure or are highly soluble in water.

It can be concluded that what we observed in this context is a particular specificity response linked to the molecular structure of analytes. This specificity indicates that amphiphilic molecules characterized by hydrophobic/aromatic regions may contribute to a more favorable response. Conversely, the sensors are unable to respond to highly hydrophilic analytes that possess significant solubility in water and lack hydrophobic/aromatic structures within their composition. Considering the molecular structures, the mechanism of the response process can be suggested as follows. The alkyl tails of the DMOAP ligand on F-SiNPs induce homeotropic/tilted anchoring of 5CB. When the specified analytes adsorb onto the nanoparticle-decorated interface, they accumulate at the DMOAP-5CB interface, causing 5CB to no longer interact exclusively with the hydrocarbon tail. Instead, it may also interact with aromatic rings, hydrophobic functional groups, or localized charged groups of the analytes. These modifications that “mask” the alkyl chains of DMOAP would subsequently shift the orientation of the LC toward a more predominant planar state. Consequently, the DMOAP-coated nanoparticle-decorated interface serves as a highly sensitive transducer when this chemical interaction is coupled with the competition between W and *F*_elastic_. Although such observations are promising, it is important to note that achieving a true selectivity, which is the focus of our ongoing studies, presents a significant challenge as it requires developing a meticulously engineered nanoparticle interface functionalized with a highly specialized ligand capable of selectively capturing target molecules from (usually challenging) mixtures.

Lastly, it is important to highlight here that a range of analytical techniques, including chromatographic, spectroscopic/elemental, optical/spectroscopic, and electrochemical methods, are employed for detecting analytes in aqueous environments. Notable examples include gas chromatography–mass spectrometry (GC-MS), atomic absorption spectroscopy (AAS), surface-enhanced Raman scattering (SERS), and electrochemical biosensors, which exhibit sensitivity ranges of 5–50 ppt, 0.01–10 ppb, ∼1 ppt, and 0.3 ppt–1 ppb, respectively.^55^ We note that our intention is not to encourage direct comparisons of our results with established methodologies; rather, we aim to enhance these techniques through our contributions to the field. As a case in point, Wang et al.^56^ have recently reported a machine learning-assisted LC droplet array platform for the sensitive and selective detection of two amphiphilic per- and polyfluoroalkyl substances (PFAS), including PFOA and perfluorooctanesulfonic acid (PFOS) in water at concentrations as low as 1 and 3.5 parts per trillion (ppt) for PFOA and PFOS, respectively. By developing an autoencoder neural network, they were able to capture the essential characteristics of LC droplet arrays treated with water samples containing various concentrations of PFOA and subsequently used the output latent space to train a basic classifier network. They showed that although low concentrations of these analytes do not lead to changes in the optical appearances of the LC droplets that are discernible or diagnostic to the trained human eye, the neural network is capable of extracting valuable information to predict their presence accurately. In terms of advancement in this research, our nanoparticle-integrated LC-based microfluidics sensors were able to (i) enhance the sensitivity limit of PFOA to 0.1 ppt, (ii) exhibit very apparent changes in the optical characteristics of LC in the vicinity of nanoparticle-decorated interface that can be identified by the naked-eye, and (iii) introduce selectivity based on the details of the chemical structures of the analytical targets.

## 3 Conclusion

This work introduced nanoparticle-integrated LC-based sensors featuring high sensitivity, facilitated through engineered soft interface continuous systems in microfluidic platforms. Using FCM images, the adsorption characteristics and positioning of DMOAP F-SiNPs at the LC–aqueous phase interface were successfully tracked. We demonstrated that decorating the LC-aqueous interface with full coverage of the interface exhibited response with a LOD of 0.1 ppb. Reducing the concentration of adsorbed nanoparticles at the interface can significantly enhance the sensor’s performance in detecting trace-level concentrations of analytes. Accordingly, the incorporation of nanoparticles at dilutions ranging from 10- to 30-fold at the interface resulted in microfluidic sensors capable of responding to ultralow analyte concentrations, achieving the LOD as low as 0.01–0.1 ppt. Such outstanding response performance, which, to our knowledge, is significantly challenging with many other reported methods, originates from the localized straining of the LC director, caused by the partial adsorption of nanoparticles at the interface along the contact line. The sensor is also powered by a considerable selectivity in detecting a range of analytes in water that do not induce an ordering transition upon direct adsorption to the LC interface from the aqueous phase. In detail, the analytes include amphiphilic molecules characterized by hydrophobic/aromatic regions. While our experimental data clearly demonstrate that the structuring of nanoparticles at the LC-aqueous interface drastically enhances sensitivity toward the target analyte, the exact underlying mechanism remains to be fully elucidated. Although we attempted to explain this enhanced performance through arguments based on elastic deformations and interfacial anchoring energies, such factors may not exclusively determine the response of the system. Further systematic variations of the nanoparticle interfaces, combined with high-sensitivity analytical tools (e.g., fluorometric or spectroscopic measurements), would be performed to fully decouple the interconnected roles of interface chemistry, anchoring, and elasticity. Nevertheless, we believe our nanoparticle-integrated LC-based microfluidic sensors are ideal candidates for a broad range of real-time or periodic monitoring applications, including the detection of micropollutants present in aquatic systems and personal diagnostics. As we showed that the selectivity of the sensor response originated from the details in the interactions of the analyte molecule and the chemistries of the nanoparticle interfaces and the mesogenic constituents of the LC phase, the next step toward developing applications would be to engineer their chemistries (both nanoparticle interfaces and LC mesogens) toward specific analytical species.

## 4 Materials and Methods

### 4.1 Materials

The primary reagents utilized in the synthesis of nanoparticles included tetraethoxysilane (TEOS), fluorescein 5-isothiocyanate (FITC), and (3-aminopropyl) triethoxysilane (APTES). Additional high-purity grade reagents and solvents employed in the synthesis process encompassed absolute ethanol, aqueous ammonia solutions (25%), cyclohexane, *n*-hexanol, and *t*-octylnonylphenol polyethoxylate ether (Triton X-100). All reagents were purchased from Sigma-Aldrich (St. Louis, MO) and were used without further purification. The room-temperature nematic liquid crystal 4-cyano-4’-pentylbiphenyl (5CB) and reactive monomer 4-(3-acryloyoxypropyloxy) benzoic acid 2-methyl-1,4-phenylene ester (RM257) were obtained from HCCH Jiangsu Hecheng Chemical Materials Co., Ltd. (Nanjing, China). HPLC-grade acetone, 2-propanol, toluene, trichloro­(octadecyl)­silane (OTS), dimethyloctadecyl­[3-(trimethoxysilyl)­propyl]­ammonium chloride (DMOAP), photoinitiator 2,2-dimethoxy-2-phenylacetophenone (DMPAP), and Nile Red dye were received from Sigma-Aldrich (St. Louis, MO). Deionized water (DW) was generated by a Heal Force water purification system, achieving a resistivity of 18.2 MΩ.cm. The positive photoresist AZ P4620, along with AZ EBR solvent, AZ 400 K developer 1:4, and buffered oxide etchant 7:1 (BOE), were sourced from Microchemicals GmbH (Ulm, Germany). Polydimethylsiloxane (PDMS) Sylgard 184 Silicone Elastomer Kit was supplied by Dow Europe GmbH (Wiesbaden, Germany). Glass slides were purchased from Marienfeld GmbH (Lauda-Königshofen, Germany). Coverslips were obtained from ISOLAB Laborgeräte GmbH (Eschau, Germany). The chemicals listed in Table 1, used as analytes in this study, were sourced as follows: Methylene blue (MB) and Methyl Orange were received from Merck. Acridine Orange was purchased from BLDpharm (Shanghai, China). Diclofenac was obtained from Acros Organics (Geel, Belgium). Indomethacin sodium hydrate was acquired from MedChemExpress (New Jersey). PFOA, ampicillin sodium salt, Ochratoxin A, erythromycin, BSA, l-leucine, l-serine, and l-aspartic acid were sourced from Sigma-Aldrich.

### 4.2 Synthesis of Core–Shell Fluorescent Silica Nanoparticles (F-SiNPs)

The fluorescent nanoparticles synthesized in this work comprise a core of the fluorophore (FITC) surrounded by a shell featuring silanol and siloxane functional groups. FITC has been reported to exhibit substantial fluorescence intensity and photostability, retaining these characteristics even after a month of exposure to white-light irradiation.^66,67^

The methodology employed for the synthesis of fluorescent SiNPs was based on the water-in-oil reverse microemulsion technique documented in the literature.^66,68,69^ Initially, a fluorescein-based silane was prepared through the reaction of FITC and APTES via the formation of a thiourea (SC­(NH)_2_) linkage. This reaction was carried out in absolute ethanol with continuous magnetic stirring for 12 h under dark conditions at room temperature. A water-in-oil microemulsion was formed by mixing cyclohexane, n-hexanol, Triton X-100, and DW, followed by stirring the mixture for 30 min. The fluorescent precursor solution was then introduced dropwise into the microemulsion under magnetic stirring for 15 min at ambient temperature. Silica polymerization was initiated through the simultaneous addition of TEOS and NH_4_OH 25% (serving as a catalyst) to the reaction mixture. Upon completion of the reaction after 20 h, the microemulsion system was disrupted by the addition of a 1:1 (v/v) DW:acetone solution, allowing for the subsequent isolation of nanoparticles from the suspension. The synthesized particles were centrifuged (10,000 rpm, 10 min) using a Hettich Universal 320 centrifuge and washed several times with ethanol, acetone, and DW to remove the surfactant and unreacted species. The resulting slurry of nanoparticles was then resuspended and preserved in DW. The amounts of materials used at various stages of the synthesis process are presented in Table 2.

**Table 2:** Specific Amounts of Chemicals Used at Each Stage of the F-SiNPs Synthesis Process

synthesis stage	chemicals	amount
fluorescent precursor solution	FITC	6 (mg)
	APTES	14.3 (mg)
	absolute EtOH	3 (mL)
microemulsion	cyclohexane	94 (mL)
	*n*-hexanol	22 (mL)
	triton x-100	22 (mL)
	DW	6.7 (mL)
reaction	NH_4_OH 25%	0.90 (mL)
	TEOS	1.2 (mL)

### 4.3 Interfacial Functionalization of F-SiNPs with DMOAP

One vol % DMOAP was added to the synthesized aqueous suspension of F-SiNPs and sonicated in an ultrasonic bath for 15 min. The DMOAP-functionalized F-SiNPs were isolated from the medium through centrifugation (10,000 rpm, 10 min). The supernatant was replaced with DW, and the particles were redispersed in the aqueous phase by using sonication. The centrifugation-redispersion process was repeated at least ten times to ensure the thorough removal of the unreacted silane molecules.

### 4.4 Preparation of Analyte Solutions

A 1 ppm stock solution of a chemical used as an analyte in the sensor response experiments was prepared by dissolving 0.5 mg of the chemical in 500 mL of ultrapure water. To prepare a 2 mL solution with a concentration of 10 ppb, 20 μL of the stock solution was diluted in 1980 μL of water. Subsequently, a 2 mL solution with a concentration of 1 ppb was prepared by taking 200 μL of the 10 ppb solution and diluting it in 1800 μL of water. Similarly, the process of serial dilution was carried out until reaching a concentration of 0.01 ppt.

### 4.5 Preparation of LC-in-Water Emulsion

To prepare LC-in-water emulsions, 3 μL of 5CB was introduced into a vial containing 1 mL of an aqueous F-SiNPs suspension. The formation of 5CB droplets was achieved by subjecting the mixture to vortex mixing for 30 s at 3000 rpm.

### 4.6 Preparation of Nanoparticle-Decorated Polymerized LC Droplets

RM257–5CB mesogen mixture was prepared by mixing 75 wt % 5CB, 25 wt % RM257, and 1 wt % photoinitiator DMPAP and homogenizing in toluene as a cosolvent, using a vortex mixture.^41^ Keeping the emulsion in the dark, toluene was allowed to evaporate naturally to obtain the reactive mesogens mixture. 3 μL of this mixture was used to prepare the LC-in-water emulsion, as explained earlier. Following the formation of the emulsion and subsequent adsorption of nanoparticles, LC droplets were polymerized under a 365 nm UV light source for 30 min. Free nanoparticles and unreacted mesogens were then removed by rinsing the emulsion at least three times with ethanol. The extraction process during rinsing was performed through natural sedimentation.

### 4.7 Characterization Techniques

Optical characterizations of LC droplet configurations were conducted using a polarized optical microscope, specifically an Olympus BX53 (Tokyo, Japan), which is equipped with a 50× objective. DLS, ζ-potential, and concentration measurements of the synthesized nanoparticles, as well as the ζ-potential analysis of LC-in-water emulsions, were carried out using a Zetasizer Ultra (Malvern Instruments Ltd., US).

The responses of the microfluidic 5CB interfaces to analytes were quantified using Fiji ImageJ, an open-source software designed for image analysis. To this end, we measured the intensity of 5CB bulk flow (*i*_ref_), as well as the mean intensities of 5CB–aqueous interface upon the decoration of the interface with the nanoparticles *(i*_NPs_ *)* and after the adsorption of analytes *(i*_A_ *)*. Applying a signal formula (eq 2) derived from a generated function based on interfacial intensities, the response *(R)* of LC to various concentrations of analytes was calculated. In this equation, *I*_A_ and *I*_NPs_ represent the differences between the corresponding interfacial intensities and *i*_ref_.

2 $$R_{\left(\mathrm{micro}\;\mathrm{fluidics}\right)}=\frac{{-(I}_{A}{-I}_{\mathrm{NPs}})}{I_{\mathrm{NPs}}}\times 100$$

The responses of LC droplet-based sensors were quantified using a generated signal formula (eq 3) that accounts for the variations in the total number of preradial and radial droplets following the decoration of 5CB droplets with nanoparticles and after the adsorption of analytes, represented as *(*Pr *+ R)*_NPs_ and *(*Pr *+ R)*_A_, respectively.

3 $$R_{\left(\mathrm{droplets}\right)}=\frac{{-[\left(\mathrm{Pr}+R\right)}_{A}{-\left(\mathrm{Pr}+R\right)}_{\mathrm{NPs}}]}{{\left(\mathrm{Pr}+R\right)}_{\mathrm{NPs}}}\times 100$$

### 4.8 Fabrication of Microchannels on Glass Slides

As shown in Figure 2a, the sketch of the channel used in the lithography process consists of two inlets and two outlet ports that combine at the main channel with 400 μm in width and 1.5 cm in length, facilitating the coflow of the nematic 5CB with an aqueous phase. Figure 9(a–f) illustrates the comprehensive fabrication procedure for microfluidic channels on glass slides. Initially, the glass slides underwent sonication in acetone and 2-propanol for 15 min each to ensure thorough cleaning. To promote hydrophobicity and enhance the attachment of the photoresist during the wet etching process, the glass slides were immersed in a 1% vol DMOAP aqueous solution and sonicated for 15 min. Subsequently, they were rinsed with water, absolute ethanol, and 2-propanol, then dried with nitrogen. The positive photoresist AZ P4620 was diluted with EBR solvent in a 2:1 ratio and applied to DMOAP-treated glass slides with a spin coater (POLOS Spin 150i, SPS-Europe B.V., Putten, The Netherlands). A uniform photoresist coating was achieved using the spin coater set to a rotational speed of 1000 rpm for 50 s, with 10 s each for linear acceleration and deceleration. The glass slides coated with photoresist were soft baked on a hot plate at 110 °C for 50 s, cooled to room temperature naturally, and allowed to equilibrate for 10 min. A maskless photolithography system, the Polos NanoWriter equipped with a 405 nm laser (SPS-Europe B.V., Putten, The Netherlands), was employed to transfer the microchannel pattern onto the photoresist-coated substrates with an exposure UV dose of 160 mJ/cm^2^. Next, the patterned glass slides were developed using the AZ 400 K developer solution for 80 s, followed by hard baking on a hot plate at 110 °C for 90 min. They were then allowed to cool to room temperature and equilibrated for at least 2 h. The isotropic wet etching process involved placing glass slides vertically in a PTFE beaker containing a 7:1 buffered oxide etch (BOE) solution. The slides were etched in an ultrasonic bath for 30 min. In order to achieve smooth and consistent etching of channel edges, the temperature of the etching process was carefully maintained not to exceed 28 °C. Afterward, the glass slides underwent a two-step rinsing process in deionized water, followed by immersion in acetone to remove the photoresist. This stripping procedure was conducted using an ultrasonic bath for 20 min. They were then promptly rinsed in succession with acetone, DW, and 2-propanol to eliminate any surface residues. Finally, the glass slides were dried with nitrogen to prepare them for glass-PDMS bonding.

**Figure 9:**
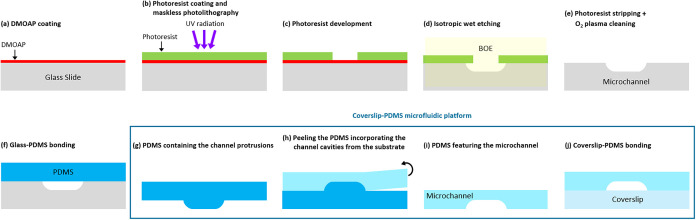
Essential steps illustrating the fabrication of microfluidic platforms. (a) DMOAP coating on a glass slide. (b) Coating of positive photoresist and maskless photolithography. (c) Photoresist development. (d) Creation of channel cavities through wet etching using a 7:1 BOE. (e) Configuration of the patterned glass slide after the photoresist stripping. (f) Preparation of the glass-PDMS microfluidic channel following the bonding process facilitated by oxygen plasma treatment. Images (g–j) represent the sequential process involved in fabricating a microchannel on PDMS, utilizing a prefabricated microchannel on a glass slide, as established in step (e). (g) Creation of the corresponding microchannel protrusions on PDMS. (h) Detaching the PDMS piece with the microchannel cavities from the PDMS substrate that contains the protrusions. (i) Configuration of the microchannel on PDMS. (j) The coverslip-PDMS microfluidic channel is prepared after bonding via oxygen plasma treatment.

To prepare PDMS, 10 wt % of curing agent was added dropwise to the elastomer base and mixed for 5 min to ensure uniformity. The mixture was subjected to a vacuum for 30 min to facilitate the removal of any trapped air bubbles. Flat PDMS molds were fabricated using aluminum foil and glass slides coated with OTS, which was deposited in a vacuum desiccator for 30 min prior to use. The transparent PDMS mixture was cast onto the prepared molds and heated for 2 h at 60 °C in an oven. Following the cooling of the molds to ambient temperature, an etched glass slide was bonded to a flat PDMS elastomer of an appropriate size, which had been prepared using a 1.5 mm biopsy punch for the inlet and outlet ports of the channels. The bonding of glass and PDMS was achieved through oxygen plasma treatment, using the Diener Electronics (Ebhausen, Germany) device. The PDMS surface and etched glass were exposed to an oxygen stream for 30 s under vacuum, with the chamber prevacuumed for 2 min. This was followed by a 20 s application of plasma and gas ventilation. Subsequently, the etched glass slide and PDMS elastomer were bonded to finalize the preparation of the microfluidic channel. The plasma treatment led to wettability alteration of the glass and PDMS surfaces to a hydrophilic state. 1.5 mm PTFE tubing was employed to facilitate the introduction of inlet materials into the channel. The tubing was integrated into a microfluidic flow control system (OB1MK3+ flow controller, ElveFlow, Paris, France).

### 4.9 Fabrication of Microchannels on PDMS

Due to the constraints associated with conducting z-stack experiments to examine the positioning of nanoparticles at the 5CB–aqueous phase interface within the microchannels, even the use of a 40× objective lens proved insufficient. Consequently, it became essential to fabricate negative microchannels on a PDMS substrate. This technique facilitated the bonding of the microchannel to a coverslip, thereby enabling the application of a 63× objective lens, which significantly enhanced observation capabilities. The sequential process involved in fabricating a microchannel on PDMS is shown in Figure 9(g–j). For this purpose, a prefabricated etched microchannel on a glass slide was selected and subjected to sonication in a 1% vol DMOAP aqueous solution for 15 min. Following this, it was rinsed with water, absolute ethanol, and 2-propanol, and then dried with nitrogen. A mold was prepared using the DMOAP-coated microchannel on the glass slide, covered with aluminum foil. A transparent PDMS mixture was cast onto the mold and cured for 2 h at 60 °C in an oven. After allowing the mold to cool to ambient temperature, the PDMS incorporating the printed protrusions of the microchannel on the glass slide was carefully excised and subsequently treated with OTS within a vacuum chamber for 30 min. The OTS-coated PDMS piece was then placed in a flat mold made from a fresh glass slide and aluminum foil. A transparent PDMS mixture was poured onto the prepared mold and heated for 2 h at 60 °C in an oven. Finally, upon reaching room temperature, the upper layer of PDMS where the microchannel cavities were formed was gently peeled from the PDMS substrate featuring the channel protrusions. The cavities of the microchannel fabricated on the PDMS exhibit symmetry with the cavities of the microchannel on the glass slide. Following the previously described methodology, the PDMS was precisely perforated to create the inlet and outlet ports and then bonded to a coverslip using oxygen plasma treatment.

### 4.10 Functionalization of the Microchannels

To enable contact between the aqueous phase and 5CB flow, the surface wetting of half of the channel was modified via DMOAP functionalization to retain hydrophobicity. For this purpose, water flow was initially introduced to the microfluidic channel platform for at least 15 min. A 1 vol % DMOAP aqueous solution was then fed to the channel from the opposite inlet. The optimal inlet pressures of water and DMOAP solution, which effectively established a stable virtual wall-flow system, were regulated at 150 and 140 mbar, respectively. The functionalization process was carried out for 30 min. Afterward, the tubing supplying DMOAP was gently detached and the entire channel was thoroughly rinsed with water for at least 15 min. Next, a flow of 5CB was introduced into the channel while maintaining water flow at a reduced pressure of 10 mbar. This approach was implemented to mitigate the risk of contamination or loss of hydrophobicity as the water flow was deliberately continued without interruption. As 5CB progressed along a specified pathway within the channel, a soft interface was established between 5CB and water.^35,36^ All microfluidic flow experiments were performed at room temperature.

### 4.11 Microscopy of Microfluidic Channel Systems

Micrographs in microfluidic experiments were captured using a Zeiss LSM 900 Fluorescence Confocal Polarizing Microscope (Jena, Germany), which features a rotatable polarizer and analyzer set at 45°–135° in the experiments. The FCPM technique was employed to ascertain the alignment of LC mesogens within the channel by acquiring confocal images at 0° and 90° polarization of the fluorescence excitation light, thereby elucidating the 3D structure. For this imaging process, the fluorescent Nile Red dye (λ_ex_ = 549 nm, λ_em_ = 628 nm) was used at a concentration of 0.01 wt % in 5CB.^70^ The *z*-stack experiments for Nile Red were performed with a 63x objective with a 5% intensity of 651 nm laser, a 53 μm pinhole, and an 800 V master gain. Further, the positioning of the nanoparticles along the channel was determined by FCM imaging. This was achieved by tracking the FITC dye molecules (λ_ex_ = 490 nm, λ_em_ = 515 nm), which are located at the core of the synthesized core–shell F-SiNPs. The *z*-stack parameters for FITC were configured to a 4.5% intensity of 488 nm laser, a 53 μm pinhole, and an 800 V master gain, using a 63× objective lens.

## Data Availability

All raw experimental data supporting the findings of this study are openly available on zenodo.org.
